# Corticothalamic Pathways in Auditory Processing: Recent Advances and Insights From Other Sensory Systems

**DOI:** 10.3389/fncir.2021.721186

**Published:** 2021-08-19

**Authors:** Flora M. Antunes, Manuel S. Malmierca

**Affiliations:** ^1^Cognitive and Auditory Neuroscience Laboratory (CANELAB), Institute of Neuroscience of Castilla y León (INCYL), University of Salamanca, Salamanca, Spain; ^2^Institute for Biomedical Research of Salamanca, University of Salamanca, Salamanca, Spain; ^3^Department of Cell Biology and Pathology, School of Medicine, University of Salamanca, Salamanca, Spain

**Keywords:** corticothalamic circuits, hierarchical inference, feedback loops, reticular thalamic nucleus, transthalamic pathways

## Abstract

The corticothalamic (CT) pathways emanate from either Layer 5 (L5) or 6 (L6) of the neocortex and largely outnumber the ascending, thalamocortical pathways. The CT pathways provide the anatomical foundations for an intricate, bidirectional communication between thalamus and cortex. They act as dynamic circuits of information transfer with the ability to modulate or even drive the response properties of target neurons at each synaptic node of the circuit. L6 CT feedback pathways enable the cortex to shape the nature of its driving inputs, by directly modulating the sensory message arriving at the thalamus. L5 CT pathways can drive the postsynaptic neurons and initiate a transthalamic corticocortical circuit by which cortical areas communicate with each other. For this reason, L5 CT pathways place the thalamus at the heart of information transfer through the cortical hierarchy. Recent evidence goes even further to suggest that the thalamus via CT pathways regulates functional connectivity within and across cortical regions, and might be engaged in cognition, behavior, and perceptual inference. As descending pathways that enable reciprocal and context-dependent communication between thalamus and cortex, we venture that CT projections are particularly interesting in the context of hierarchical perceptual inference formulations such as those contemplated in predictive processing schemes, which so far heavily rely on cortical implementations. We discuss recent proposals suggesting that the thalamus, and particularly higher order thalamus via transthalamic pathways, could coordinate and contextualize hierarchical inference in cortical hierarchies. We will explore these ideas with a focus on the auditory system.

## Introduction

A massive set of glutamatergic corticothalamic projections arising from the pyramidal cells in Layers 5 (L5) or 6 (L6) of the cortex outnumber the ascending, thalamocortical projections and inextricably link the cortex to the thalamus (Kelly and Wong, 1981; Sherman and Guillery, 1998; Winer et al., 2001; Harris et al., 2019). These CT projections are not homogeneous but differ anatomically and functionally paving the way to different modes of interaction between thalamus and cortex in both ways. The small but numerous terminals of upper L6 (L6a) CT axons, together with their collaterals to the thalamic reticular nucleus (Ojima, 1994; Rouiller and Welker, 2000; Hazama et al., 2004), send a reciprocal feedback to the thalamus and are known to modulate the sensory message arriving at the thalamus in a myriad of ways (Yu et al., 2004; Zhang and Yan, 2008; Guo et al., 2017; Homma et al., 2017). In contrast, the giant terminals carried by L5 axons (Bajo et al., 1995; Bartlett et al., 2000) can drive their own messages to thalamic neurons *via* non-reciprocal projections, and then feedforward these messages to a different, hierarchically higher cortical area, forming a transthalamic corticocortical circuit (Llano and Sherman, 2008; Theyel et al., 2010; Mo and Sherman, 2019). Very recent studies suggest that some CT neurons emanating from deep layer 6 (L6b) have distinct anatomical and physiological properties from neurons emanating from both L6a and L5 and could represent a third CT circuit (Hoerder-Suabedissen et al., 2018; Ansorge et al., 2020; Buchan, 2020; Zolnik et al., 2020).

Altogether, these different CT circuits enrich and diversify the opportunities for bidirectional communication between thalamus and cortex. At the thalamic node, CT circuits actively transform and/or gate the transmission of sensory information en route to the cortex (Yu et al., 2004; Antunes and Malmierca, 2011; Mease et al., 2014; Cai et al., 2016; Guo et al., 2017; Homma et al., 2017; Ibrahim et al., 2021) but also regulate functional connectivity within and across cortical areas (Saalmann et al., 2012; Sherman, 2016; Zhou et al., 2016; Schmitt et al., 2017; Jaramillo et al., 2019). CT circuits expand the computational capabilities of the thalamus, reflecting its active role in sensory processing and beyond (Nakajima and Halassa, 2017; Rikhye et al., 2018). It is now widely accepted that the thalamus and CT pathways are engaged in high level computations previously thought to be exclusively cortical, such as language (Bartlett, 2013; Llano, 2013; Crosson, 2019; Mihai et al., 2019), learning and memory (Wolff and Vann, 2019), attention (Zhou et al., 2016; Schmitt et al., 2017), behavioral flexibility (Nakayama et al., 2018), and perceptual decision making (Halassa and Sherman, 2019). Recent evidence suggests that CT pathways may play a role in sensory attenuation of self-generated stimuli (Hua et al., 2020; Clayton et al., 2021) and perceptual inference (Bastos et al., 2012; Kanai et al., 2015; Auksztulewicz and Friston, 2016; Rikhye et al., 2018; Asilador and Llano, 2020).

In the following, we first review the circuitry, physiology, and function of CT projections (section L5 and L6 Corticothalamic Projections Provide Different Inputs to Thalamic Neurons). Then, we will discuss the participation of L5 CT projections in transthalamic pathways (section L5 Corticothalamic Projections Initiate Transthalamic Corticocortical Pathways). Finally, we propose that the thalamus and the CT pathways participate in the coordination and contextualization of hierarchical inference in cortical hierarchies (section Role of Corticothalamic Pathways in the Implementation of Predictive Processing Frameworks) (Mumford, 1991; Kanai et al., 2015; Rikhye et al., 2018).

## L5 and L6 Corticothalamic Projections Provide Different Inputs to Thalamic Neurons

**Figure 1:**
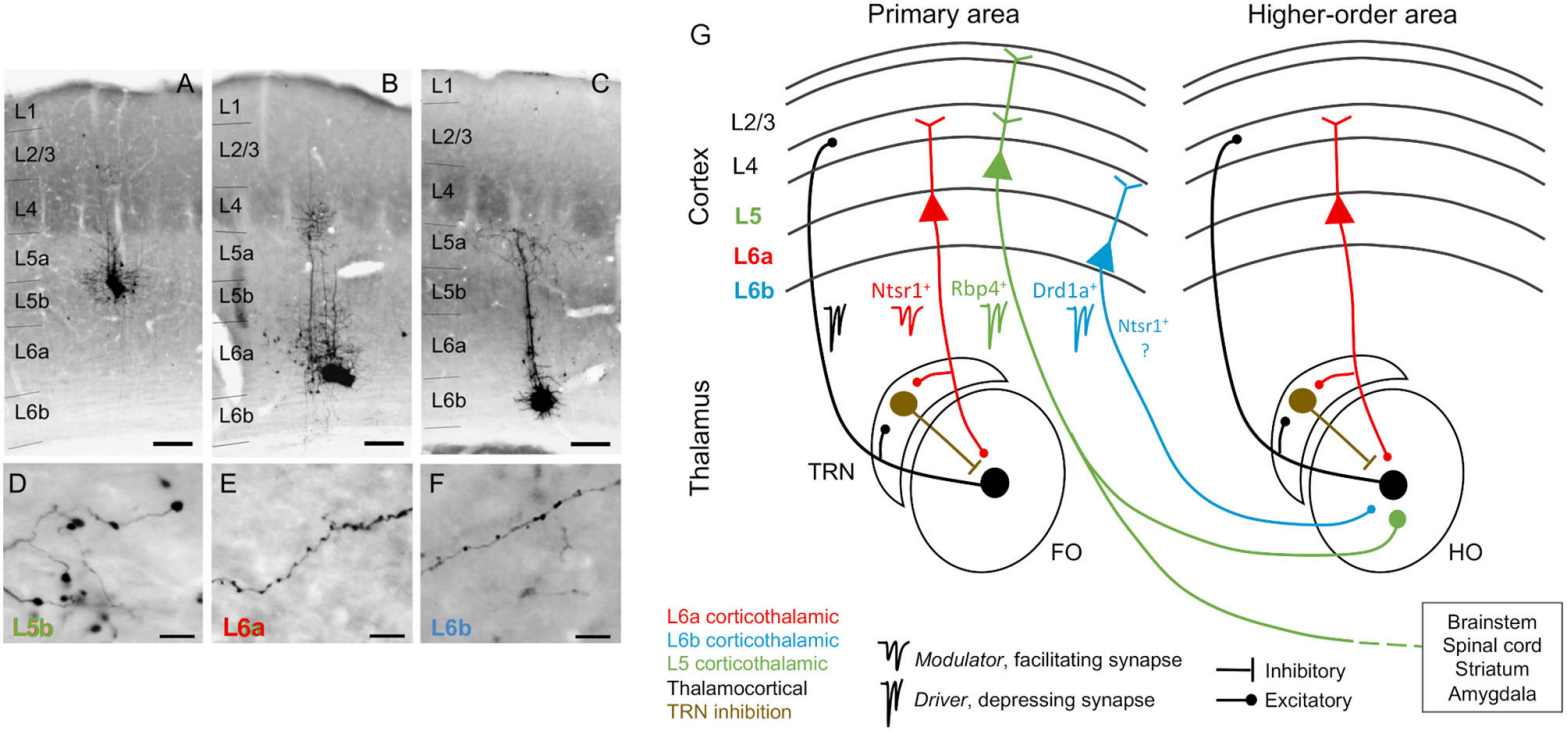
Corticothalamic neurons and circuits. **(A–C)** Photomicrographs showing BDA microdeposits in L5b, L6a, and L6b neurons of primary somatosensory barrel field **(D–F)**, and their respective axonal arborizations in (higher order) posterior thalamic nucleus. Cortical pyramidal neurons differ in the morphology of their axonal varicosities. Scale bars: A–C = 150 μm, D–F = 10 μm. **(G)** Schematic and simplified view of corticothalamic and thalamocortical circuits, based on information from several sensory systems (auditory, visual, and somatosensory). Red, L6a CT projections, *modulator* (Ntsr1^+^): feedback projections that send collaterals to the TRN, a GABAergic nucleus that provides inhibition to the thalamus (brown). Black, thalamocortical, feedforward projections that form reciprocal loops with L6a feedback projections. Green, L5 CT projections, *driver* (Rbp4^+^): project non-reciprocally to a hierarchically higher order thalamic nucleus and form part of transthalamic corticocortical pathways; they are collaterals from long-range axons that project to other subcortical centers (e.g., brainstem, spinal cord, striatum, and amygdala). Blue, L6b CT projections, *driver* (Drd1a^+^): project non-reciprocally to a hierarchically higher order thalamic nucleus; it is unknown if Drd1a+ and Ntsr1+ neurons in L6b are overlapping or distinct neuronal populations. As insets, examples of specific markers from Cre mouse lines that can be used to selectively target neurons of each CT circuit [Ntsr1-Cre, Drd1a-Cre, and Rbp4-Cre mice, for L6a (red), L6b (blue), and L5 (green) CT neurons]. FO, first order thalamic nuclei; HO, higher order thalamic nuclei; TRN, thalamic reticular nucleus; L2/3, cortical layers 2 and 3; L4, cortical layer 4; L5, cortical layer 5; L6a, cortical layer 6a; L6b, cortical layer 6b; Photomicrographs **(A–F)** after Hoerder-Suabedissen et al. (2018).

In this review, we will adopt the conceptualization proposed by Sherman and Guillery (1998) in which glutamatergic pathways can be divided into *drivers* and *modulators*. Drivers are the main conduits of information and strongly activate the postsynaptic neuron, whereas modulators serve to modify the processing of information carried by driver inputs without changing the basic nature (such as the receptive field shape) of the message to be relayed. In this context, L5 CT projection provides driver input to thalamic neurons, similarly to the ascending, feedforward inputs whereas L6 cortical feedback modulates thalamic relay neurons, performing similar operations as the classical neuromodulators do (e.g., acetylcholine, noradrenaline, serotonin; Usrey and Sherman, 2019). Thalamic nuclei that receive subcortical driver inputs are referred to as first *order* and represent the first sensory input to cortex, whereas nuclei that receive driver influence from cortical L5 are referred to as *higher order* and represent part of a corticothalamocortical, or transthalamic, pathway that conveys information from one cortical area to another (Sherman, 2016). In the auditory thalamus, they correspond to the ventral (MGV) and dorsal (MGD) subdivisions of the MGB, respectively (Ojima, 1994; Llano and Sherman, 2008; Lee and Murray Sherman, 2010). According to this model, first *order* nuclei (e.g., MGV) receive a reciprocal feedback input from L6 but no input from L5, whereas *higher order* nuclei (e.g., MGD and MGM) receive two distinct cortical inputs: one from L6 that is a reciprocal feedback, and another from L5 that is non-reciprocal (Figure 1G; Llano and Sherman, 2008; Usrey and Sherman, 2019). In this review, we consider as feedforward connections all bottom-up connections with driver properties, and feedback connections all top-down connections with modulatory properties. According to this classification, and from a cortical perspective, we consider L6 reciprocal connections as top-down feedback connections whereas L5 non-reciprocal connections as bottom-up feedforward connections within transthalamic pathways.

Recent studies that selectively targeted and manipulated distinct neuronal subtypes in L6 suggest that the previous classification might be incomplete, because not all L6 CT neurons send a reciprocal feedback to thalamus or provide modulator-like input. A subpopulation of neurons in L6b seems to be involved in a CT circuit that differs anatomically and physiologically from both L5 and L6a, the canonical L6 feedback circuit, and likely represent a third type of CT circuit (Figure 1; Hoerder-Suabedissen et al., 2018; Ansorge et al., 2020; Buchan, 2020; Zolnik et al., 2020), as we will explain in section L6b non-reciprocal Corticothalamic Projections.

### L6a Corticothalamic Feedback Forms a Corticothalamic Loop

Feedback signals to thalamus from cortex arise from pyramidal neurons in the upper layer 6 (L6a; Bourassa and Deschênes, 1995; Thomson, 2010; Malmierca, 2015). These feedback neurons can be genetically targeted using expression of the type 1 neurotensin receptor (Ntsr1- Cre transgenic mice; Olsen et al., 2012; Bortone et al., 2014; Guo et al., 2017). L6a CT neurons specifically target the region of sensory thalamus from which they receive direct input, and their dendrites and ascending collaterals target L4, the major thalamorecipient layer, thus preserving thalamocortical-corticothalamic reciprocal connectivity (Figure 1). This forms a thalamocortical loop, by which thalamus and cortex concurrently stimulate (and are stimulated by) each other (Bajo et al., 1995; Zhang and Deschênes, 1997; But see Kim et al., 2014, who report that L6 CT neurons strongly innervate, and excite, pyramidal neurons in layer 5). For example, L6 from primary auditory cortex (A1) projects to tonotopically comparable laminae of the same subdivision from which it received its main thalamocortical input, the MGV (Winer et al., 2001; Hazama et al., 2004; Llano and Sherman, 2008). The L6 A1-MGV projections constitutes one of the largest feedback pathways in the auditory system (Rouiller and Welker, 1991; Ojima, 1994; Prieto and Winer, 1999; Kimura et al., 2005). Similar reciprocal connectivity occurs between the MGD and secondary auditory cortex (A2). L6 feedback axons are composed of thin fibers having small but numerous glutamatergic boutons that synapse on distal dendrites and evoke facilitating EPSPs *via* ionotropic and metabotropic receptors (Figure 1; Ojima, 1994; Bajo et al., 1995; Winer et al., 2001; Bartlett and Smith, 2002), leading to their characterization as modulators (Sherman and Guillery, 1998).

#### The Corticothalamic Loop Engages the GABAergic Thalamic Reticular Nucleus

In their way to thalamus, L6 axons send collaterals to the TRN. The TRN is a thin shell of GABAergic neurons surrounding the thalamus that projects to the same thalamic nucleus (but not exclusively) as the L6 fibers passing through it (Figure 1; Crabtree, 1998; Pinault, 2004; Kimura et al., 2005). When the TRN is activated by L6 collaterals, it provides feedforward inhibition to the MGB. For this reason, the passage of L6 collaterals by the TRN determines to great extent the modulatory effect exerted by L6 excitatory terminals on MGB neurons (Guillery, 1995; Deschênes et al., 1998; Kimura et al., 2012). The excitatory or inhibitory sign of L6 CT modulation will depend on a delicate balance between a prevalent effect exerted on TRN-mediated disynaptic inhibition or direct, monosynaptic CT excitation (Crandall et al., 2015; Li and Ebner, 2016; Guo et al., 2017). For example, low frequency thalamocortical oscillations that occur during slow-wave sleep depend on rhythmic inhibition of thalamocortical neurons. This rhythmic inhibition is likely caused by a stronger CT effect on disynaptic inhibition that overcomes monosynaptic excitation (Golshani et al., 2001; Steriade, 2001).

The intricate and delicate corticothalamic interactions together with the fact that the TRN is a very small nucleus closely adjacent to the thalamus, both lying deep in the brain, makes the study of the relative contribution of disynaptic inhibition and direct excitation in CT modulation difficult to disentangle. A study using brain slices that preserved L6 CT circuitry has shown a dynamic excitatory-inhibitory balance shift in thalamic excitability that depended on the rate and time-course of L6 CT activation (Crandall et al., 2015). Thalamic excitability was mainly suppressed during low frequency CT activity, whereas it shifted to facilitation following higher frequency CT activity. The shifting to facilitation was the result of facilitation of monosynaptic CT-evoked EPSCs (as expected, because L6 feedback projection is facilitatory), together with a reduction of CT-triggered disynaptic IPSCs (*via* TRN). This reduction was due to short-term synaptic depression of the TRN-thalamus synapse, with a minimal contribution from the intrinsically mediated reductions in TRN spiking (the intrinsic burst properties of TRN neurons cannot follow high frequencies; Crandall et al., 2015). A recent study in the auditory system investigated the mechanisms underlying the gating of all-or-none population responses in the auditory cortex *via* L6 CT-TRN feedback (Ibrahim et al., 2021). They present an alternative mechanism by which the gating of cortical activity mediated by L6 CT-TRN feedback is dependent on the ability of the TRN to desynchronize thalamocortical neurons rather than diminishing thalamic activity (Ibrahim et al., 2021). They suggest that thalamic synchronization by the TRN can be a mechanism to recruit neuronal populations for sensory representations (Ibrahim et al., 2021).

In summary, the TRN node empowers the CT circuits with the ability to flexibly change functional connectivity by acting as a regulator that can favor or oppose the relay of sensory information to the cortex as required by ongoing behavioral demands (Kimura et al., 2012; Li and Ebner, 2016; Guo et al., 2017), for example, during sleep (Steriade and Deschenes, 1984; Golshani et al., 2001; Barthó et al., 2014), sensorimotor processing (Marlinski et al., 2012) or attention (Crick, 1984; McAlonan et al., 2008; Wells et al., 2016).

#### Thalamic Modulation by CT Feedback Is Difficult to Disambiguate From Classical Studies

Classical studies in the intact brain used techniques to silence (Ryugo and Weinberger, 1976; Orman and Humphrey, 1981) or stimulate (Watanabe et al., 1966; Aitkin and Dunlop, 1969) entire cortical regions without discriminating between cortical layers or accounting for the effects that this non-specific manipulation could have on subthalamic regions that may themselves provide inputs to the thalamus. Largely because of these limitations, the general view of corticothalamic interactions from classical studies is one of very large variability, with divergent effects. These effects are often difficult to interpret in terms of perception and behavior.

**Figure 2:**
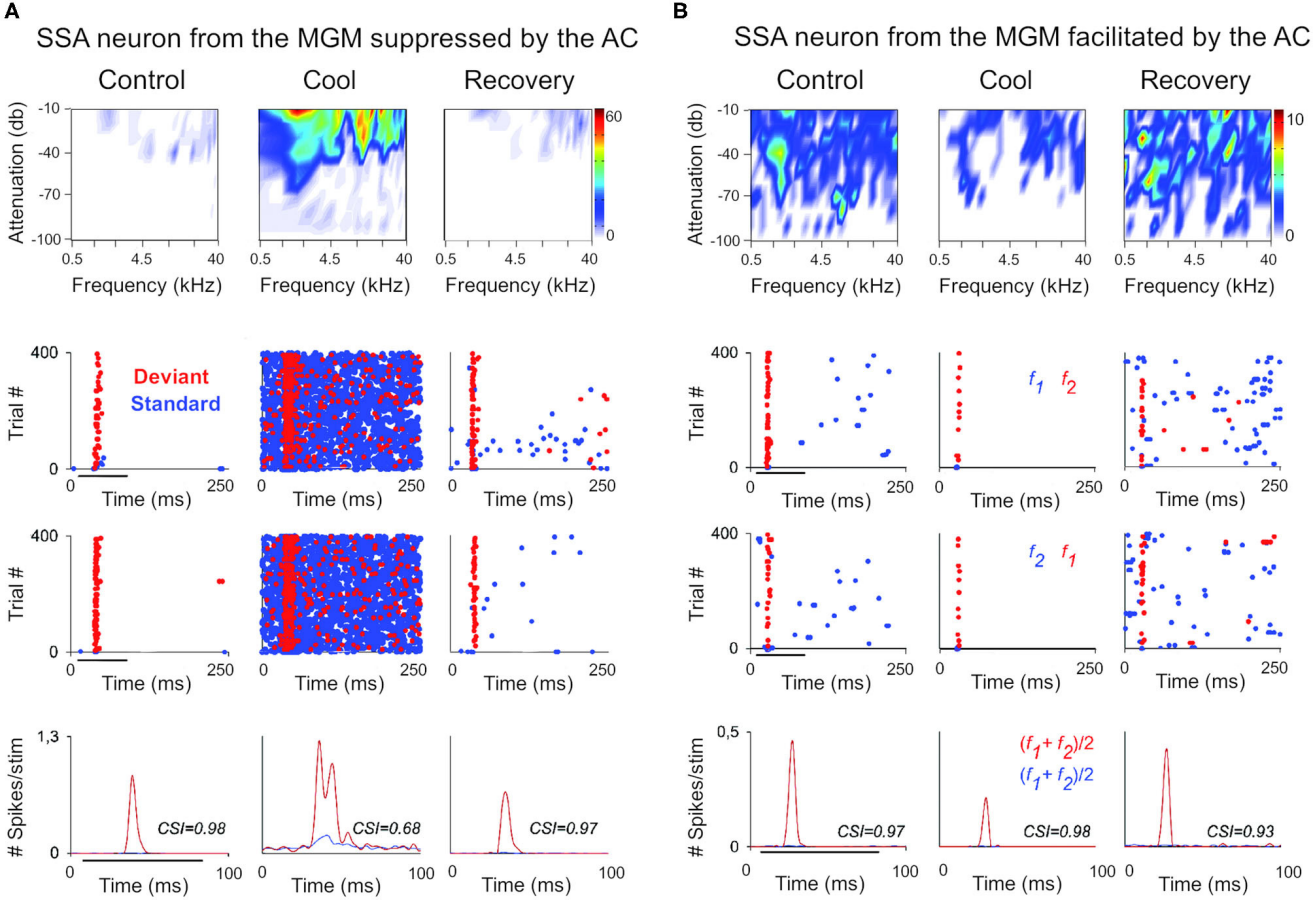
The cortex exerts suppressive and facilitating influences in neurons of the auditory thalamus. Examples of single-unit responses to auditory stimulation in the MGB of the anesthetized rat before, during, and after AC deactivation by cooling. **(A)** A neuron localized to the MGM that receives suppressive influences from the AC. The frequency response areas (first row), and the responses of the neuron to the oddball paradigm (second-fourth rows), in the control, cool and recovery conditions. The oddball paradigm was used to elicit SSA in these neurons. Briefly, the oddball paradigm consisted of a sequence of a repetitive stimulus (standard; 90% probability) that was infrequently interrupted by a different stimulus (deviant; 10% probability. The standard (blue) and the deviant (red) stimulus were pure tones selected from within the frequency response area of the neuron. Two blocks of 400 trials each (middle panels) were presented in which the standard and deviant frequencies were reversed (second panel, first block: f1/f2 as standard/deviant; third panel, second block: f2/f1 as standard/deviant). Dot rasters show individual spikes to the deviant and standard (red and blue dots, respectively), in the three conditions for the two stimulus presentation blocks (stacked along the y-axis; repetition rate 4 Hz; stimulus duration: 75 ms, black horizontal lines under the plots). PSTHs (last row) show the number of spikes/stimulus (bin duration: 3 ms) averaged over the two blocks [(f1+f2)/2; blue line is standard, red line is deviant]. The CSI calculated for each condition is noted as an inset on the PSTHs. The CSI quantifies the amount of SSA and is calculated as CSI = [d(f1)+d(f2)-s(f1)-s(f2)]/[d(f1)+d(f2)+s(f1)+s(f2)], where d(fi) and s(fi) are responses (# spikes/stimulus) to frequency fi when deviant or standard, respectively (0 ≤ CSI ≤ 1). Higher CSI values, higher SSA. **(B)** Responses of another neuron localized to the MGM that receives facilitatory influences from the AC, presented as in **(A)**. AC, auditory cortex; MGB, medial geniculate body; MGM, medial subdivision of the medial geniculate body; SSA, stimulus specific adaptation; CSI, common SSA index; f1, frequency 1; f2, frequency 2. Adapted from Antunes and Malmierca (2011).

**Figure 3:**
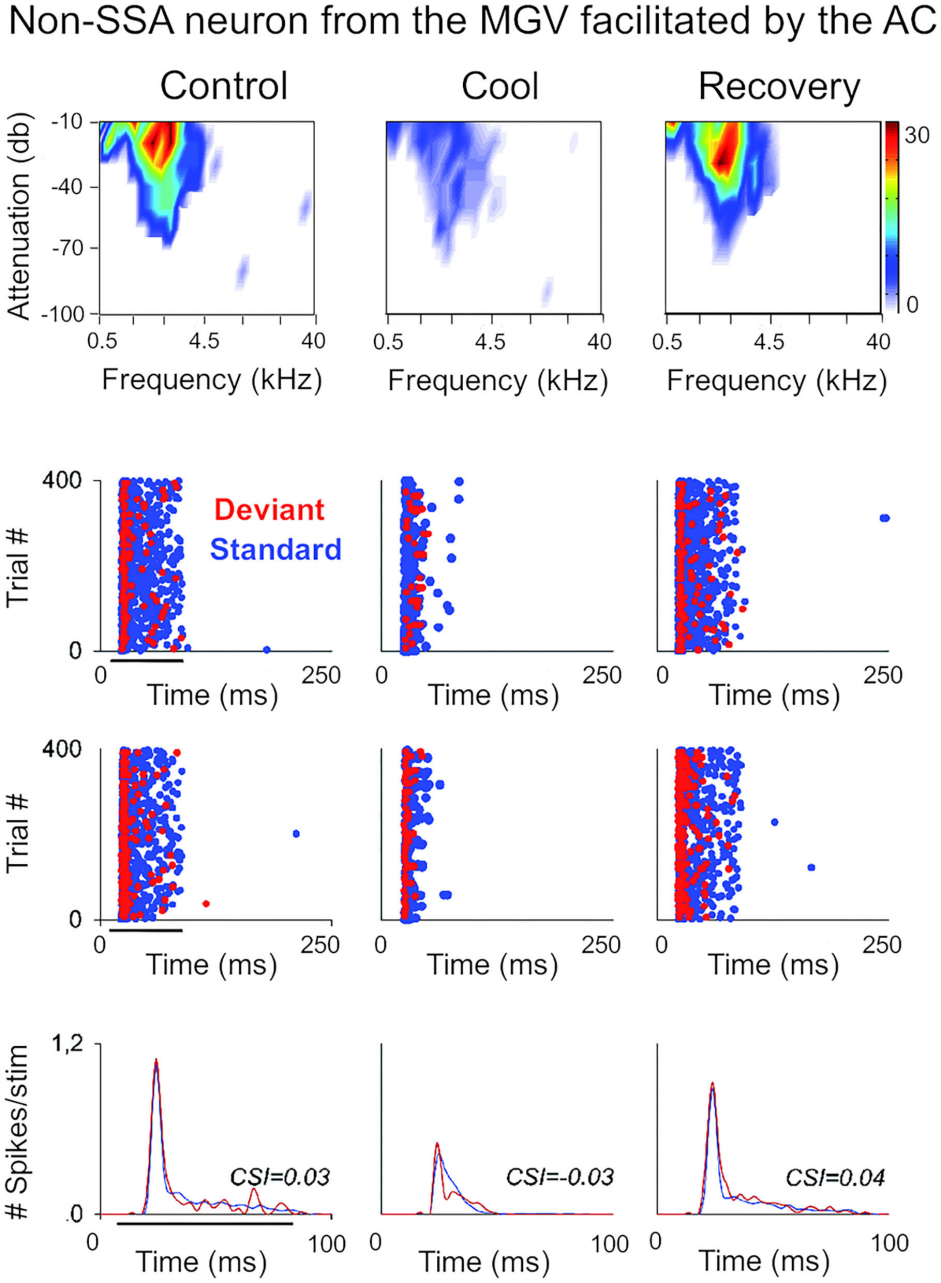
Non-SSA neurons primarily receive facilitatory influences from the AC. Example of a neuron recorded from the MGV that was facilitated by the AC, presented as in Figure 2. The neuron responds consistently to both the standard and the deviant over the trials, i.e., it does not show SSA as confirmed by the low CSI value (~0). The CSI value was not significantly changed by AC deactivation. AC, auditory cortex; MGV, ventral subdivision of the medial geniculate body; CSI, common SSA index. Adapted from Antunes and Malmierca (2011).

**Figure 4:**
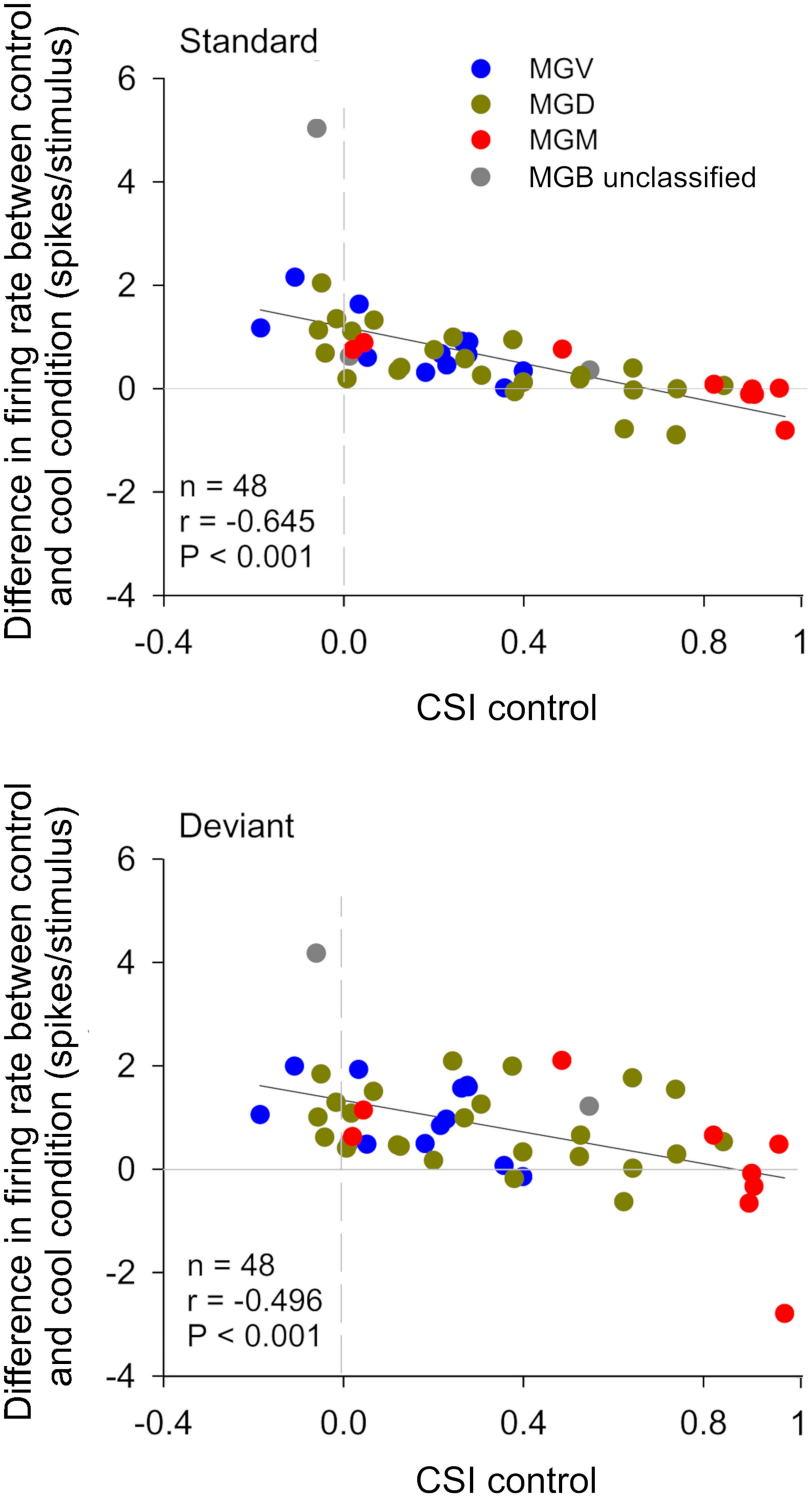
The gain exerted by the cortex in auditory thalamic neurons depends on their ability to signal a deviance from previous stimulation context. Scatterplots of the CSI (control condition) vs. the difference in firing rate between the control and cool conditions (spikes/stimulus difference) in response to the standard (upper panel) and deviant stimulus (lower panel), for each neuron. Blue, green, and red dots represent the neurons that were localized to the ventral (*n* = 12), dorsal (*n* = 24), and medial (*n* = 9) subdivisions of the MGB, respectively (*n* = 45, neurons that were localized to one of the three MGB subdivision). Gray dots represent MGB neurons that were not localized to a specific MGB subdivisions (*n* = 3). In both plots, positive values (above the horizontal line at the origin) indicate a reduction in firing rate with cortical deactivation (neurons receive facilitatory cortical influences), whereas negative values (bellow the horizontal line) indicate an increment in firing rate with cortical deactivation (neurons receive suppressive influences from the cortex). The difference in firing rate was inversely correlated with CSI for both standard and deviant stimuli. The slopes of the standard and deviant regression lines are not significantly different from each other [ANCOVA: main effect of stimuli, *F*_(1,92)_ = 1.89, *p* = 0.172; main effect of CSI, *F*_(1,92)_ = 43.27, *p* = 0; interaction, *F*_(1,92)_ = 0.23, *p* = 0.634; *n* = 48], indicating that the correlation coefficients between standard and deviant are not different. The AC differentially affects the discharge rate of neurons depending on their SSA level. Neurons without SSA are mainly facilitated, whereas some neurons with high SSA are suppressed by the cortex. AC, auditory cortex; MGB, medial geniculate body; MGV, ventral subdivision of the MGB; MGD, dorsal subdivision of the MGB; MGM, medial subdivision of the MGB; SSA, stimulus specific adaptation; CSI, common SSA index; Adapted from Antunes and Malmierca (2011).

In the auditory system, cortical inactivation (Villa and Abeles, 1990; Villa et al., 1991; Zhang et al., 1997; Palmer et al., 2007) and/or stimulation (He, 2003; Xiong et al., 2004; Yu et al., 2004; Zhang and Yan, 2008; Ojima and Rouiller, 2011) experiments have demonstrated that the AC can modulate the MGB either by facilitation or by suppression, resulting in changes in receptive field properties and firing patterns. Indeed, a previous study corroborates these findings by showing that the basic properties of MGB neurons (e.g., spontaneous activity, discharge rates, latencies) were altered during cortical silencing by cooling (Figures 2, 3; Antunes and Malmierca, 2011). However, stimulus-specific adaptation (SSA), the property that we were investigating in this study was not altered during cortical silencing (Figure 2). SSA measures the neuronal adaptation to repeated sounds (standards) that does not generalize to rare sounds (deviants; Ulanovsky et al., 2003). In fact, responses to deviant sounds are enhanced following the repetition, and consequent adaptation, of the standard sound (Parras et al., 2017). For this reason, these neurons are believed to be context-sensitive: they signal a deviance from the previous context (repetition of a stimulus). It was shown that SSA is implemented at the MGB level largely independently of cortical activity (Figure 2; Antunes and Malmierca, 2011). Contrast adaptation, a type of adaptation that is also dependent on stimulus statistics, has also been demonstrated to occur in auditory thalamus independently of cortical activity (Lohse et al., 2020). Regarding SSA, the auditory cortex modulates the discharge rate of MGB neurons affecting similarly the responses to the standard and the deviant stimuli, probably by providing a gain-control mechanism (because the amount of SSA is quantified by a ratio of driving rates, it is largely unaffected by cortical silencing; Figures 2, 3). But perhaps the most interesting finding of Antunes and Malmierca (2011) study was the fact that the gain exerted on MGB neurons depended on the level of SSA that they exhibited: high SSA was related to weaker cortical gain (Figure 4). Because this relationship is not dependent on the subdivision to which the MGB neurons belong, this finding provided strong evidence for a clear rule that relates cortical modulation to a neuronal property (Antunes and Malmierca, 2011). We will speculate about the possible mechanism underlying this SSA-dependent CT modulation of MGB neurons on section Role of CT Pathways in Coordinating and Contextualizing Inference in Cortical Hierarchies. Altogether, our results are consistent with the idea that the CT feedback scales the sensitivity of MGB neurons to its driving inputs by controlling their gain (Villa and Abeles, 1990; Villa et al., 1991; He, 2003; Mease et al., 2014). Such gain control might improve coding of low salience stimuli (Cai et al., 2016), promote detection or discrimination of behaviorally relevant stimuli (Happel et al., 2014; Guo et al., 2017; Homma et al., 2017), mediate sound-specific plastic changes in thalamic neurons (Zhang and Yan, 2008; Nelson et al., 2015), and underly auditory attention (He, 2003).

#### Selective Manipulation of L6 CT Pathways Disentangles Their Roles in Auditory Perception and Behavior

Recent studies using a combination of layer or cell-type specific selective manipulation, electrophysiology and behavior testing have just started to unravel the roles of CT pathways in behavior and perception (Guo et al., 2017; Nakayama et al., 2018; Clayton et al., 2021; Ibrahim et al., 2021; Lohse et al., 2021). For example, Homma et al. (2017) used chromophore-targeted laser photolysis to selectively eliminate the input from layer VI to the MGV. In their study, the authors provided behavioral evidence that L6 CT—MGV feedback pathway contributes to the perception of complex sounds, by showing that this pathway is needed for the normal ability of ferrets to detect a mistuned harmonic within a complex sound. Since normal hearing uses deviations from harmonicity to segregate concurrent sounds, L6 CT feedback may play a role in auditory scene analysis (Homma et al., 2017). In humans, task-dependent modulation of the MGV (but not the other MGB subdivisions) facilitates speech recognition performance (fMRI study; Mihai et al., 2019). Such modulation might be provided by L6 CT feedback, as Mihai et al. (2019) suggested, although this hypothesis needs further confirmation. Happel et al. (2014) demonstrated that dopaminergic modulation regulates a corticothalamocortical positive-feedback circuit in A1 that boosts horizontal intracortical processing (long range corticocortical networks) and enhances the detection of behaviorally relevant stimuli.

**Figure 5:**
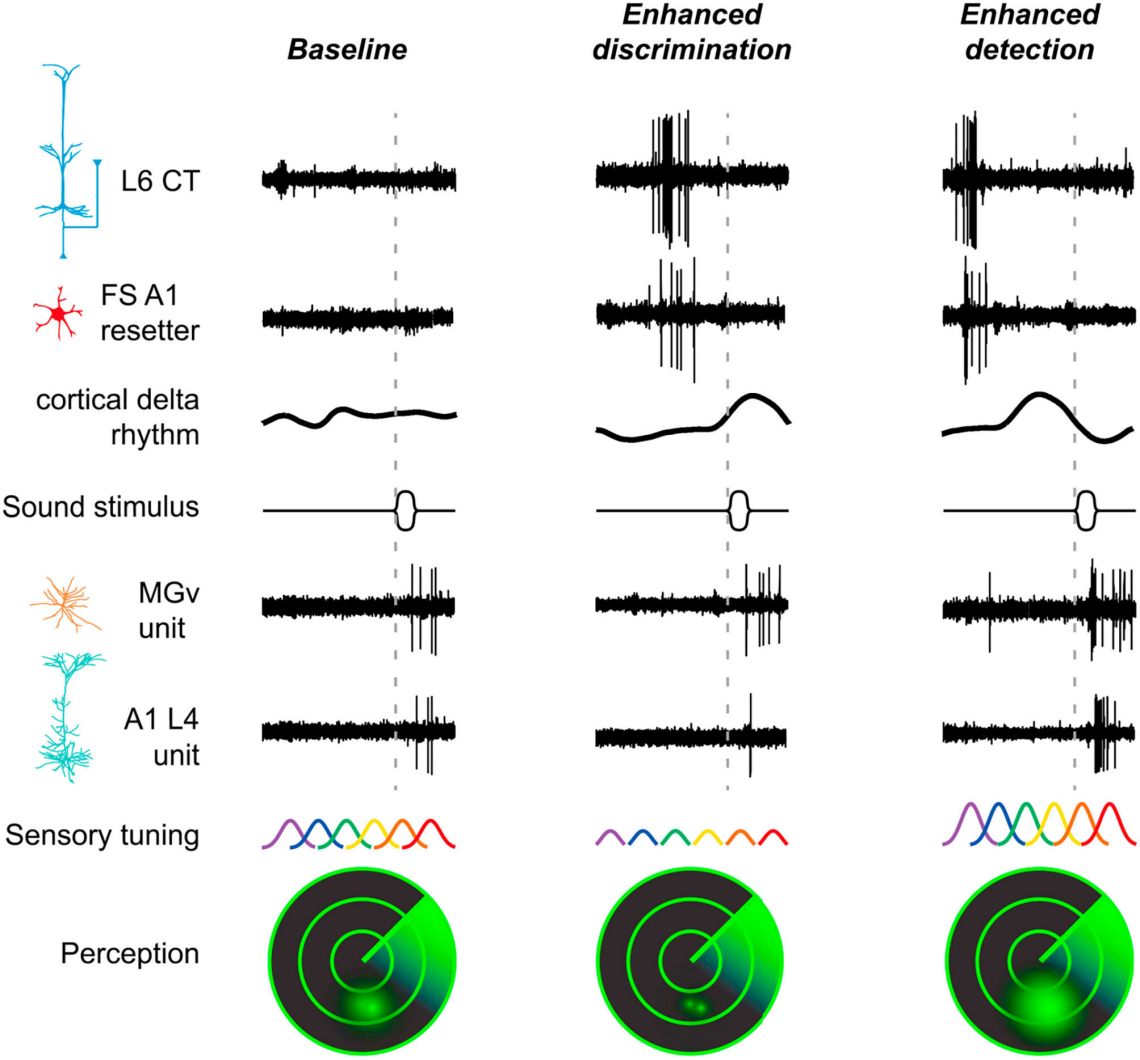
L6 CT neurons are involved in the behavioral switch between sound detection and discrimination. This scheme summarizes the findings by Guo et al. (2017) who demonstrate the participation of L6 CT neurons (Ntsr1+), via both their intracortical and corticothalamic connections to perceptual modes of enhanced detection or discrimination. Left column, in a baseline condition with low activity in L6 CT neurons and fast-spiking interneurons (FS resetters), the sound-evoked activity in MGV and L4 cortical neurons is moderate. FS resetters are activated following intense firing of L6 CT neurons. Activity of FS resetters increases the power and resets the phase of low frequency rhythms. Middle column, at a short delay period following intense activity of L6 CT and FS resetter neurons, the delta-theta rhythm is at a positive, low excitability phase, and sound-evoked activity is suppressed in A1 but not in the MGV, which favors tone discrimination at the expense of sound sensitivity (the reduced excitability of cortical neurons sharpens frequency tuning). Right column, at longer delays following L6 CT activity, the phase of the cortical rhythm has rotated to a negative, high excitability phase, and sound-evoked activity is enhanced both in A1 and MGV. The enhanced excitability of cortical neurons is expected to increase the overlap in sensory tuning between neighboring tuning regions, which favors tone detection at the expense of reduced tone discrimination. A1, primary auditory cortex; CT, corticothalamic; L6, cortical layer 6; L4, cortical layer 4; MGV, ventral subdivision of the medial geniculate body; FS, fast-spiking interneurons. Reproduced, with permission, from Guo et al. (2017).

L6 CT neurons not only send a feedback to the thalamus but also have dense intracortical connections with both excitatory and inhibitory neurons throughout the cortical column (Zhang and Deschênes, 1997; Winer et al., 2001; Llano and Sherman, 2008; Williamson and Polley, 2019). This means that activation of L6 CT neurons could modulate both thalamocortical transmission and intracortical processing (Olsen et al., 2012; Bortone et al., 2014; Guo et al., 2017), producing mixed excitatory and inhibitory effects on both thalamic and cortical neurons (Temereanca and Simons, 2004; Mease et al., 2014; Denman and Contreras, 2015). A recent study in the auditory system demonstrated that spontaneous and sound-evoked activity of A1 in awake mice was enhanced during optogenetic activation of L6 CT neurons [Ntsr1+; Guo et al. (2017)]. Interestingly, this study went further to investigate whether activity in A1 and thalamus in the awake animal could also depend on timing between L6 CT activation and sensory stimulation, similarly to what occurs in barrel cortex slices (the sign of CT modulation depends on frequency and timing of CT activation; Crandall et al., 2015). This is indeed the case in the auditory system of the awake animal (Guo et al., 2017): at short delays following offset of L6 CT activation, tone-evoked responses in A1 were suppressed but more precisely tuned, whereas at long delays, responses were enhanced but less precisely tuned (Figure 5, compare middle to right panels). Noteworthily, this bidirectional modulation serves as a behavioral switch, favoring either tone discrimination or detection (Figure 5). The ability to discriminate between similar stimuli is favored in the short delay period following L6 CT activation, whereas the ability to detect faint sounds is favored in the long delay period.

Intense firing of L6 CT neurons activates a subpopulation of fast spiking (FS) cortical interneurons (Olsen et al., 2012; Bortone et al., 2014). Activation of FS interneurons increases the power and resets the phase of low-frequency oscillations, a mechanism identified by Guo et al. (2017) that might explain the differences found (Figure 5). In brief, cortical suppression and improved sound discrimination following offset of L6 CT activation arise from induction of the early, low excitability phase of an intracortical delta-theta rhythm. This rhythm is reset in the short delay period by the FS resetters (Figure 5). In contrast, cortical enhancement and improved tone detection in the long delay period arise primarily from an intra-thalamic shift in excitatory-inhibitory balance between MGV and TRN, where disynaptic inhibition is scaled down over time, similarly to that described by Crandall et al. (2015).

As Linden (2017) noted, the question remains as to whether the low-frequency phase reset mechanism unraveled by Guo et al. (2017) has a role during active listening. During active listening, entrainment of low-frequency oscillations to the attended auditory stream is believed to enhance neuronal responses and perception (Zion Golumbic et al., 2013; Obleser and Kayser, 2019). This entrainment ensures that local neurons are in the high excitability phase of the oscillations when relevant inputs arrive, so they can be forwarded up the hierarchy. In contrast, sensory inputs from the non-attended stream will arrive at the low excitability phase of the oscillations, and will be suppressed (Schroeder and Lakatos, 2009; Zion Golumbic et al., 2013). Recently, it was shown that L6 CT neurons can be activated by motor-related input prior to anticipated sounds during active sensing (Clayton et al., 2021), which might contribute to sensory attenuation of self-generated sounds as demonstrated in humans (Hua et al., 2020).

In summary, the prominent feedback projections from cortical L6 to thalamus have intrigued auditory scientists since many decades, as displayed by the numerous studies dedicated to investigating their role in thalamic function. However, as we have reviewed above, L6 CT projections cannot be studied with classical techniques that are blind to the complexity of the circuits to which they belong. In recent years, the interest in meticulously studying these circuits and their roles in auditory processing and behavior has increased tremendously. This was largely propelled by the advent of new techniques that now enable the selective manipulation of L6a projections in the awake behaving animal. These studies have just started to unveil the powerful roles L6a feedback circuits have in auditory perception and behavior.

### L6b Non-reciprocal Corticothalamic Projections

Some CT neurons emanate from deep layer 6 (L6b) and have long been known to primarily innervate higher order thalamic nuclei (Figure 1; visual and auditory system; Bourassa and Deschênes, 1995; Llano and Sherman, 2008). However, the connectivity as well as behavioral and functional roles of L6b CT neurons are largely unknown. Like most L6a CT neurons, some CT neurons in L6b also express Ntsr1 (visual and somatosensory system; Olsen et al., 2012; Chevée et al., 2018). For this reason, using Ntsr1-Cre mice to manipulate L6 CT neurons, possibly engages the circuits of layers 6a and 6b. However, Ntsr1 neurons seem to have different projection patterns depending on their laminar position within L6 (visual and somatosensory system, Kim et al., 2014; Chevée et al., 2018; Frandolig et al., 2019). Ntsr1 neurons in layer 6a are thought to project exclusively reciprocally (e.g., from AI to MGV), and their apical dendrites to innervate cortical L4 to form a corticothalamic loop (see section L6a Corticothalamic Feedback Forms a Corticothalamic Loop). In contrast, Ntsr1 neurons in L6b can branch to project to first- and higher-order thalamic nuclei, although preferentially to higher order nuclei (e.g., from AI to MGD; Llano and Sherman, 2008), and their dendrites are believed to innervate cortical L5 (somatosensory system; Chevée et al., 2018; Frandolig et al., 2019), a cortical output layer where some long-range axons with collaterals to higher order thalamic nuclei emanate, as we will discuss bellow (section L5 Corticothalamic Projections).

**Figure 6:**
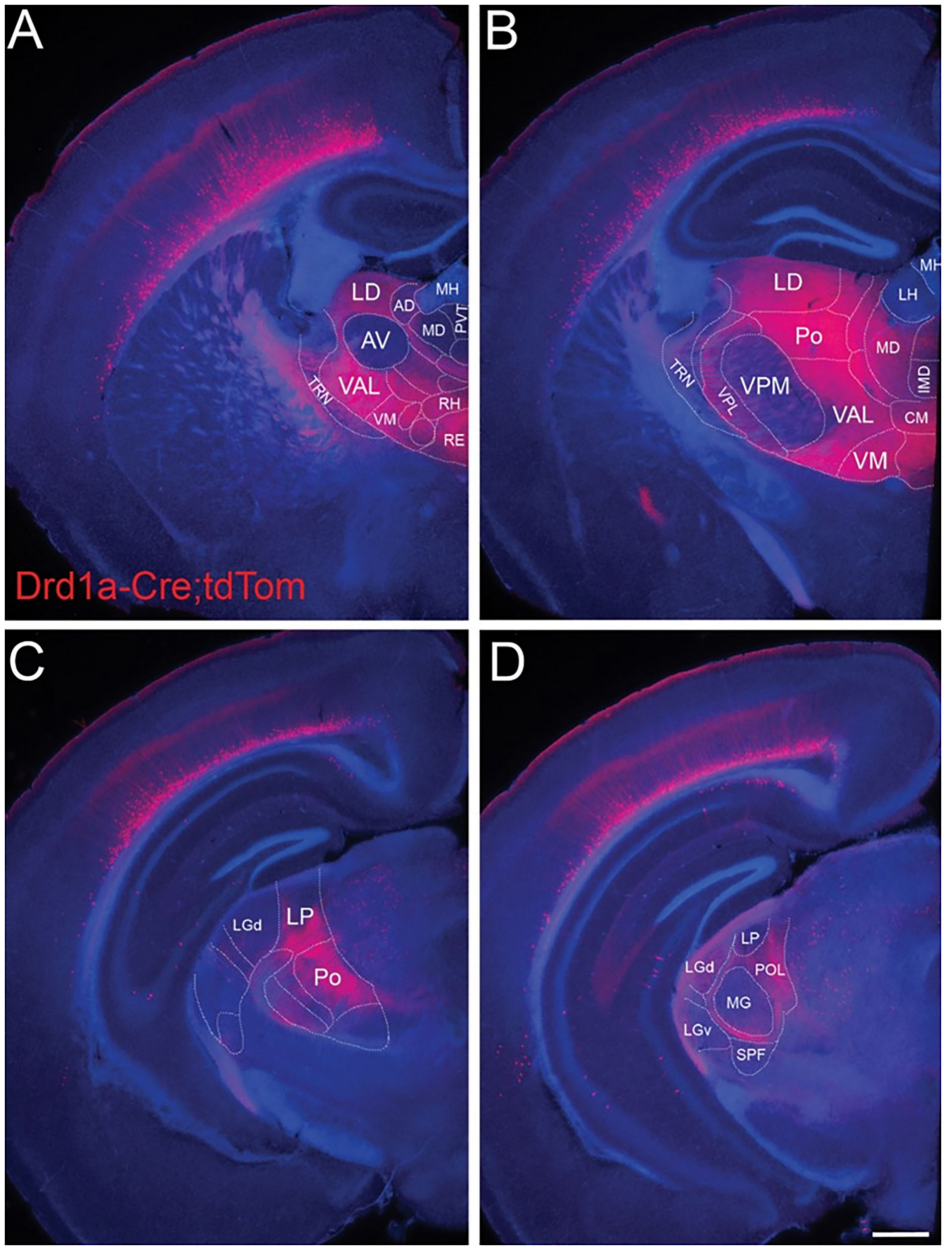
L6b neurons project to higher order and avoid first order thalamic nuclei. Drd1a-Cre expression in fibers arising from neurons in L6b of the entire cortical mantle in adult mice (P35) visualized by tdTomato labeling (Drd1a-Cre::tdTom+ fibers). Projections from L6b avoid first order auditory and non-auditory thalamic nuclei such as **(A)** the anteroventral nucleus, **(B)** the ventral-posterior medial nucleus, **(C)** the lateral geniculate nucleus anteriorly, and **(D)** the MGV in the auditory thalamus. In contrast, they innervate heavily higher order thalamic nuclei such as the lateral dorsal nucleus, the posterior nucleus, the ventral medial nucleus, and the ventral anterior lateral complex. Fibers pass through TRN without apparent branching. Scale bar Scale bar = 500 μm. AD, anterodorsal nucleus; AV, anteroventral nucleus; CM, central medial nucleus; IMD, intermediodorsal nucleus; LD, lateral dorsal nucleus; LH, lateral habenula; LGd, dorsal lateral geniculate nucleus; LGv, ventral lateral geniculate nucleus; LP, lateral posterior nucleus; MD, mediodorsal nucleus; MG, medial geniculate nucleus; MH, medial habenula; Po, posterior nucleus; POL, posterior limiting nucleus; PVT, paraventricular nucleus; RE, nucleus reuniens; RH, rhomboid nucleus; SPF, subparafascicular nucleus; TRN, thalamic reticular nucleus; VAL, ventral anterior lateral complex; VM, ventral medial nucleus; VPL, ventral-posterior lateral nucleus; VPM, ventral-posterior medial nucleus. Reproduced from Hoerder-Suabedissen et al. (2018).

The fact that using Ntsr1-Cre mice to manipulate Ntsr1 neurons engages neurons from both sublayers 6a and 6b makes it difficult to disambiguate their distinct roles and connectivity. Recently, it has become possible to selectively target a subpopulation of neurons in L6b using expression of the type 1a dopamine receptor (Drd1a; Drd1a-Cre transgenic mouse; somatosensory system; Zolnik et al., 2020). It is still unknown if Drd1a neurons are a subpopulation of Ntsr1 neurons or belong to distinct population. Ongoing research in motor, somatosensory and visual cortex targeting Drd1a neurons is unraveling key connectivity and functional differences between layers 6a and 6b projections, indicating that they are engaged in different circuits (Figure 1). These studies confirmed that L6b CT neurons strongly innervate L5 of the cortex and preferentially target higher order thalamic nuclei (somatosensory system; Zhang and Deschênes, 1997; Ansorge et al., 2020; Zolnik et al., 2020) but form few side branches or synapses in first order thalamic nuclei (e.g., the MGV of the auditory thalamus; Figure 6D; Hoerder-Suabedissen et al., 2018). Perhaps the most surprising findings were that L6b Drd1a neurons receive their main input from long range intracortical neurons (e.g., from motor, auditory, and visual regions) with little or no contribution from thalamic input (Zolnik et al., 2020), and do not have side branches or synapses in TRN (Hoerder-Suabedissen et al., 2018), as opposed to L6a neurons (Figure 1). Functionally, Drd1 neurons seem to carry a driver-like signature, like L5 CT neurons, because their synapses are depressing (somatosensory system; Ansorge et al., 2020; Buchan, 2020), although L6b axon terminals are significantly smaller and simpler than the majority from L5 axons [somatosensory system, posterior thalamic nucleus; Figure 1, compare photomicrographs in plots D and F; Hoerder-Suabedissen et al. (2018)].

Altogether, these findings suggest that L6b is positioned outside the canonical corticothalamocortical loop (Figure 1). Their connectivity to cortical L5 and higher order thalamic nuclei, regions that are associated with cognitive functions, suggests that Drd1a neurons have a role in cognition. One possibility is that these neurons participate in transthalamic connections with higher order cortical areas, similarly to L5 CT neurons. Because neurons in L6b and higher order thalamic nuclei are responsive to the wake-promoting neuropeptide orexin, it has been suggested that L6b CT neurons might be recruited in an arousal dependent manner (Hoerder-Suabedissen et al., 2018; Zolnik et al., 2020). Future studies using awake, behaving animals are needed to unveil the roles L6b neurons might have in brain state control and cognition.

### L5 Corticothalamic Projections

CT projections from cortical layer 5 are collateral projections issued from long-range axons that project to other subcortical regions in the brainstem and/or the spinal cord (Figure 1; auditory, motor, somatosensory and visual systems; Kelly and Wong, 1981; Deschênes et al., 1994; Prasad et al., 2020). In the auditory system, L5 collaterals produce few but thick axons with large glutamatergic terminals that synapse on proximal dendrites of higher order thalamic nuclei *via* ionotropic but not metabotropic receptors and do not innervate the TRN (Rouiller and Welker, 1991; Ojima, 1994; Bajo et al., 1995; Winer et al., 1999; Hazama et al., 2004; Rovó et al., 2012). However, global mappings of L5 terminals across multiple thalamic and extrathalamic sites revealed that there is a considerable variation in size of L5 terminals, ranging from small to giant terminals (entire macaque thalamus; Rovó et al., 2012), varying with cortical area of origin and target (afferents originating from somatosensory, visual, motor, and prefrontal cortex; Prasad et al., 2020). It is still unknown if small and large L5 terminals have different physiological properties and if this variation occurs in the auditory system.

L5 terminals evoke large and fast EPSCs that can trigger action potentials in the thalamic postsynaptic neurons (somatosensory system; Reichova and Sherman, 2004; Groh et al., 2008), leading to their classification as drivers (Sherman and Guillery, 1998). L5 giant synapses are not always in a driver transmission mode, because they undergo frequency-dependent short-term synaptic depression (due to their high initial Pr) by which activity is reduced during repeated presynaptic firing (e.g., spontaneous activity; lateral posterior nucleus: Li et al., 2003; posterior medial nucleus: Groh et al., 2008). In other words, due to synaptic depression, driver synapses are expected to act as low-pass filters that are most effective at transmitting impulses at the onset of presynaptic activity (Abbott and Regehr, 2004). This form of synaptic plasticity could allow switching the gating mode of L5 CT giant synapses from a dominant driver (following periods of silence) to a coincidence detector (when L5B depressed neurons fire synchronously, for example after a sensory stimulus; Groh et al., 2008). This has been demonstrated for L5B synapses onto neurons of the posterior medial nucleus (POm), a higher order thalamic nucleus of the whisker somatosensory system (Groh et al., 2008).

*In vivo* studies from the whisker system of the anesthetized mouse support the driving role of L5 corticothalamic projections (Diamond et al., 1992; Groh et al., 2014), by showing a robust transfer of spikes from a few active L5B cortical inputs to the POm (Mease et al., 2016). Interestingly, CT gain at these synapses is not constant, but it is controlled by global cortical up and down states (Mease et al., 2016). CT gain is maximal at the beginning phase of the up state but then declines during the up state due to frequency-dependent adaptation (possibly due to synaptic depression), resulting in periodic high- vs low-gain oscillations (Mease et al., 2016). Because higher order somatosensory thalamus projects to various cortical areas, single or synchronized spikes of a few L5B neurons can be amplified *in vivo* at the CT driver synapse and broadcast *via* thalamus simultaneously to motor, primary, and secondary sensory cortical regions (Deschênes et al., 1998; Theyel et al., 2010), to enhance and prolong cortical responses (Mease et al., 2016).

#### Anatomical and Physiological Evidence for L5 Driver Terminals in the Auditory System

In the auditory system, electrophysiology studies focusing on synaptic transmission of L5 terminals-MGD synapses are largely missing (but see Williamson and Polley, 2019, who recorded extracellularly L5 CT neurons in auditory cortex). This contrasts with the abundance of anatomical studies that undoubtedly show the driver-like properties of L5 terminals synapsing on neurons in MGD (Bajo et al., 1995; Rouiller and Welker, 2000; Llano and Sherman, 2008). Overall, L5 axons resemble in their structure and synaptic contacts the ascending, driving inputs that carry sensory messages to the thalamus (Sherman and Guillery, 1998). As Bajo et al. (1995) noted, the giant L5 corticothalamic boutons are reminiscent of the large auditory nerve endings, the so-called endbulbs of Held, that innervate cells in the ventral cochlear nucleus (Ramón y Cajal, 1904), as well as the calyces of Held in the medial nucleus of the trapezoid body (Held, 1891). These auditory nerve endings are known to provide highly secure synapses, with large and fast EPSCs that show strong activity-dependent synaptic depression due to their large probability of vesicle release (Pr; Schneggenburger et al., 1999; Antunes et al., 2020), similarly to L5 driver synapses. Given their anatomical similarities to the driver terminals in the visual and somatosensory systems, it is tempting to speculate that L5 projection should evoke large, depressing EPSCs mediated by ionotropic receptors without the contribution of metabotropic receptors (Li et al., 2003; Reichova and Sherman, 2004; Lee and Sherman, 2011). Furthermore, the burst-firing mode of L5 CT neurons in auditory cortex enables the transmission of a highly secure signal well-suited for a driver synapse (Llano and Sherman, 2009). Consistent with this, a few neurons (4 out of 24) recorded *in vivo* from the MGD of anesthetized rats had their sound-evoked responses eliminated during reversible cortical deactivation, suggesting that these responses were inherited from L5 driver axons (Antunes and Malmierca, 2011). However, this study deactivated the entire auditory cortex making it impossible to disentangle the effects of L5 or L6 on the observed thalamic responses. *In vivo* and *in vitro* studies manipulating specific layers and regions of the auditory cortex are needed to confirm that L5 terminals effectively behave as drivers of auditory thalamic activity, following the rule of the other sensory systems studied so far.

## L5 Corticothalamic Projections Initiate Transthalamic Corticocortical Pathways

The message conveyed by L5 to the thalamus is then feedforwarded to the cortex to form a non-reciprocal corticothalamocortical circuit by which activity in a lower order cortical area is distributed, *via* the thalamus, to a higher order cortical area (visual, somatosensory and motor system; Kato, 1990; Theyel et al., 2010; Sherman, 2016; Mo and Sherman, 2019). Llano and Sherman (2008) provided anatomical evidence that L5 CT projections emanating from A1 are endowed with the properties necessary to initiate such a transthalamic circuit. L5 of A1 projects non-reciprocally to the MGD *via* driver-like terminals (Rouiller and Welker, 1991; Ojima, 1994; Bartlett et al., 2000), and then route this information to A2 (Llano and Sherman, 2008). Theyel et al. (2010) showed that these transthalamic pathways can effectively transfer information between somatosensory cortical areas. They used flavoprotein autofluorescence *in vitro* to demonstrate that stimulation of L5B, but not L6, in primary somatosensory cortex drove robust activity in higher-order somatosensory cortex, *via* corticothalamocortical pathway activation (Theyel et al., 2010). Information transfer between primary and secondary areas continued even after permanent disruption of the direct corticocortical afferents connecting them and was only interrupted by chemically induced thalamic inhibition (Theyel et al., 2010).

### Transthalamic Pathways Can Connect Functionally Distinct Cortical Areas

The existence of transthalamic pathways paralleling the direct, hierarchical corticocortical pathways, is not restricted to communication circuits between primary and secondary auditory, visual, and somatosensory areas, but can be a more general principle bridging functionally distinct cortical areas, similar to what occurs in most higher order nuclei that receive connections from and project to multiple cortical areas (Mo and Sherman, 2019; Zajzon and Morales-Gregorio, 2019; Lohse et al., 2021). Optogenetic stimulation of a small local cluster of L5 primary visual cortical neurons in the *in vivo* mouse brain is sufficient to initiate and propagate corticothalamic Ca2+ waves as a global neuronal wave of activity *via* long-range corticothalamic integration (Stroh et al., 2013).

**Figure 7:**
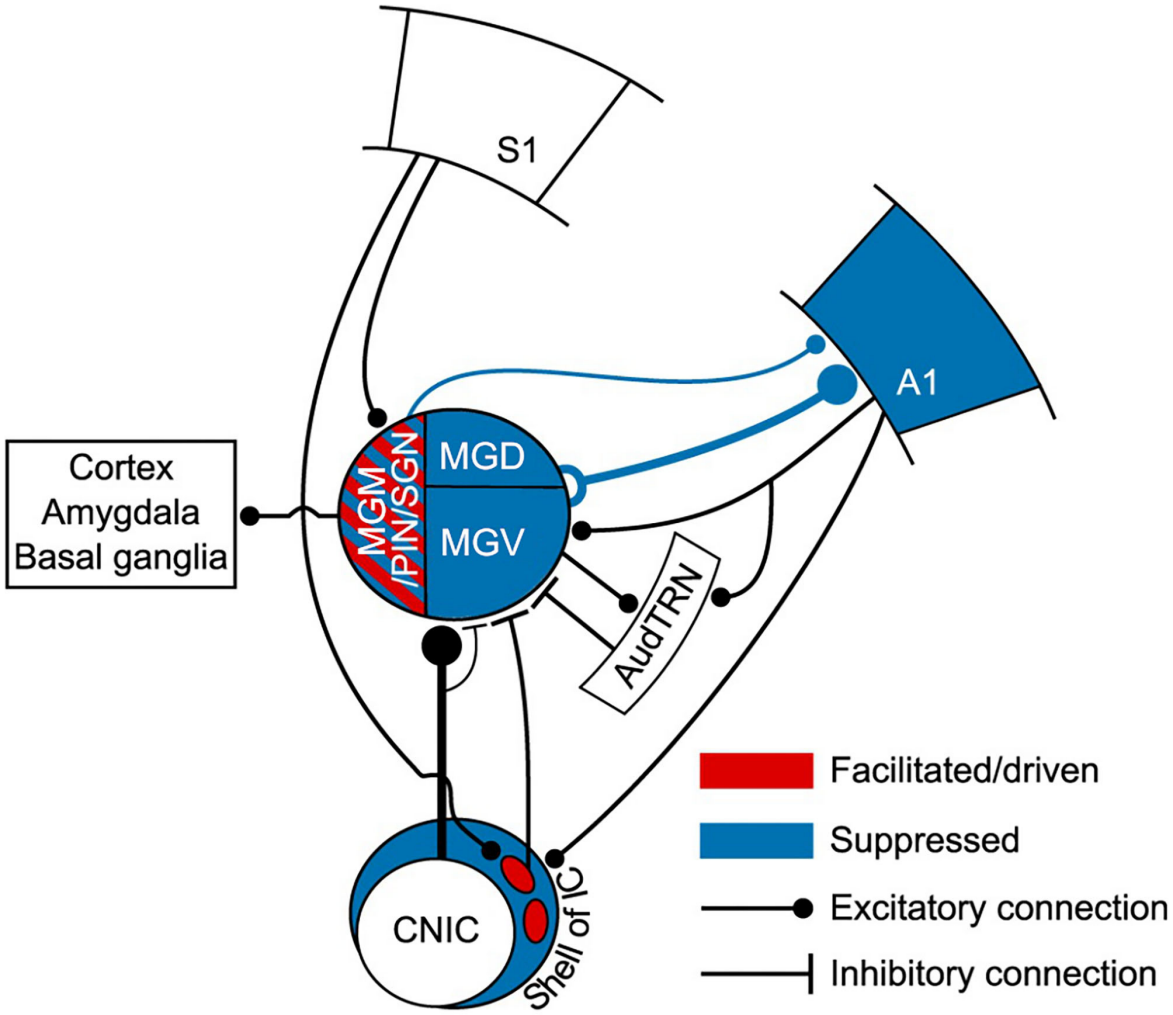
Auditory thalamus and midbrain bridging primary somatosensory cortex (S1) to primary auditory cortex (A1). This scheme summarizes the findings of Lohse et al. (2021) study. This study elegantly undisclosed the multisensory circuits by which the primary somatosensory cortex controls activity in the thalamocortical system in mice. In blue, regions of the auditory system (midbrain, thalamus and A1) that were suppressed to auditory stimulation (tones) by concurrent whisker stimulation (whisker deflection). Suppression occurred via a descending connection from S1 to neurons in the lateral shell of the inferior colliculus that project to the MGB. This resulted in a suppression of thalamocortical neurons and suppression of auditory activity in A1. Altogether, this forms a corticocolliculo-thalamocortical multisensory circuit by which somatosensory information exerts a dominance over auditory processing in A1. In red, some neurons in the medial sector of the auditory thalamus, including the MGM, had their auditory responses enhanced or driven with whisker stimulation. A direct connection arising in L5 of S1 to the medial sector of the MGB could mediate this enhancement. Because the MGM projects to cortical areas, including A1, this could form a transthalamic circuit bridging S1 to AC. However, this hypothesis needs further confirmation. A1, primary auditory cortex; AudTRN, auditory sector of the thalamic reticular nucleus; CNIC, central nucleus of the inferior colliculus; MGM/PIN/SGN, medial subdivision of the MGB/posterior intralaminar nucleus/suprageniculate nucleus; MGD, dorsal subdivision of the MGB; MGV, ventral subdivision of the MGB; S1, primary somatosensory cortex. Adapted from Lohse et al. (2021).

In the auditory system, Lohse et al. (2021) unraveled a multisensory circuit by which activation of primary somatosensory cortex by whisker stimulation suppresses responses to auditory stimuli in A1, which is implemented by a crossmodal circuit connecting somatosensory cortex, *via* auditory midbrain, to A1-projecting MGB neurons (corticocolliculo-thalamocortical circuit; Figure 7). This study demonstrates a clear role for the auditory thalamus, midbrain and descending connections in bridging cortical areas that belong to different sensory systems, as an alternative to direct corticocortical pathways (Lohse et al., 2021). It is unknown, however, if L5 is involved in the corticocolliculo-thalamocortical circuit. However, a direct projection from L5 in somatosensory cortex to the MGM exists and has been shown to facilitate responses to auditory stimuli in the MGM (Lohse et al., 2021), a nucleus that projects to other cortical areas (Figure 7; for a review see Bartlett, 2013).

Strong evidence that L5 transthalamic pathways can bridge distant and functionally distinct cortical areas comes from a study by Mo and Sherman (2019). They demonstrated that a transthalamic circuits exists between the primary somatosensory cortex and the primary motor cortex through the POm. This sensorimotor circuit is initiated in L5 primary somatosensory cortex and shows driver properties at both the corticothalamic and thalamocortical synapses. The demonstration that the primary motor cortex is involved in a transthalamic non-reciprocal circuit just like the sensory cortices, challenged previous ideas that higher order cortices would diverge from this model, with connections instead organized in reciprocal loops (Svoboda and Li, 2018; Collins and Anastasiades, 2019). Work by Guo et al. (2018) reveals that in fact this distinct organization in higher-order motor regions exists, where neurons in the ventromedial thalamus receive L5 and L6 inputs from the same high order cortical region, the anterolateral motor cortex, forming two parallel reciprocal loops. However, the study by Mo and Sherman (2019) shows us that these reciprocal cortical loops are not the only way by which higher order cortices (at least the primary motor cortex) interact with thalamic nuclei *via* L5 pyramidal neurons. The interesting discussion initiated by Collins and Anastasiades (2019) is far from being over. Most likely, the complexity of corticothalamocortical circuits will continue to surprise us as more studies come to light.

### Evidence for Convergence of Cortical and Subcortical Driver Inputs in Higher Order Thalamic Neurons

A largely unresolved and very interesting question is whether thalamic processing in higher order thalamus involves significant integration of information from convergent driver inputs. While it is generally assumed that integration occurs at cortical level rather than at the thalamus (Sherman, 2017), very interesting evidence exists that L5 and subcortical driver inputs converge and interact onto single neurons in higher order somatosensory thalamus (POm; Groh et al., 2008, 2014). Such convergence occurs when both cortical and thalamic driver inputs are active, leading to non-linear responses driven by the coincidence of these inputs within a well-defined time window, similarly to a “AND-gate” response (in an AND-gate the output equals the binary product of the inputs, meaning that a cell can only transfer the combination of the two inputs; Groh et al., 2014; Ahissar and Oram, 2015). This evidence proposes an alternative model by which thalamic neurons act as integrators of the sensory and cortical information they receive, and it is this integrated activity that they transfer back to the cortical network (Groh et al., 2014; Ahissar and Oram, 2015). Anatomical evidence suggests that the convergence of cortical and subcortical driver afferents is not widespread through the thalamus but is restricted to well-defined thalamic territories at the boundaries of first- and higher-order territories (e.g., border regions of the ventrolateral nucleus with the lateral pulvinar; Rovó et al., 2012).

The model proposed by Groh et al. (2014) entails a powerful control of ascending sensory information at the level of the thalamus by cortical L5: a strong instructive cortical signal summates with sensory information. Thus, a sensory stimulus can be fundamentally changed in the thalamus by L5 descending cortical signals and thus be perceived differently by the cortex (Groh et al., 2014). In this scenario, the role of the transthalamic pathway at the thalamic junction gains another dimension, by incorporating information from the ascending, sensory pathway before feedforwarding information to a higher order cortical region. Hypothetically, this could be a strategy for enhancing behaviorally relevant environmental features. It might be interesting in the context of model-based inferences, such as those implicated in predictive processing framework (Rao and Ballard, 1999; Friston, 2005). These theoretical frameworks rely on comparisons between prior information—in the form of prediction- and incoming sensory information (Friston, 2005; Bastos et al., 2012). In this context, the convergence of L5 CT signals with sensory information in higher order thalamic neurons (Groh et al., 2014), would enable these neurons to extract possible relationships and/or discrepancies between cortical and peripheral information (as contemplated in predictive processing frameworks; Friston, 2005; Kanai et al., 2015; discussed in section Role of Corticothalamic Pathways in the Implementation of Predictive Processing Frameworks).

### The Transthalamic Pathway Is Not Just a Relay of Information Between Cortical Regions

The view of transthalamic pathways (and the thalamus) as higher order circuits that relay information between cortical regions is now recognized to be incomplete. Multiple lines of evidence suggest that the thalamus operates as a master regulator of functional cortical connectivity within and between cortical areas *via* transthalamic pathways (Nakajima and Halassa, 2017; and perhaps concurrently with feedback pathways, Jaramillo et al., 2019). By such regulatory power, the thalamus can modulate attention (Saalmann et al., 2012; Zhou et al., 2016; Schmitt et al., 2017), impact language processing (Crosson, 2019), participate in working memory and encode confidence during decision making (Komura et al., 2013; Jaramillo et al., 2019). Considering that the thalamus receives abundant projections from subcortical, cortical and neuromodulator regions, thalamic circuits seem to be uniquely suited to provide contextual modulation to cortical computations associated with cognition (Rikhye et al., 2018; Wolff and Vann, 2019). Using computational modeling, Jaramillo et al. (2019) aggregates these ideas that altogether sustain that higher order thalamus *via* corticothalamic pathways participates in major cognitive functions and is implicated in psychiatric disorders. Furthermore, they show that transthalamic and feedback pathways (concurrently) participate in frequency-dependent inter-areal interactions that modify the relative hierarchical positions of cortical areas (Saalmann et al., 2012; Zhou et al., 2016; Jaramillo et al., 2019).

The extent to which signal transmission in the cortex is routed *via* transthalamic pathways is unknown (Sherman, 2016). The studies that we have reviewed above support the notion that higher order thalamus can use task-dependent contextual information to shape cortical responses (Kanai et al., 2015; Jaramillo et al., 2019). Because of the computational capabilities of the thalamo-cortical circuit, Jaramillo et al. (2019) suggests that thalamic nuclei predominantly modulate cortical computations. It is possible that circuits dedicated to encoding contextual information cohabit with circuits relaying information (or other computations) in the same thalamic nucleus. The utilization of these circuits according to behavioral demands can underlie attention, working memory and decision-making (Rikhye et al., 2018).

### Evidence for Top-Down Transthalamic Pathways

An interesting possibility is that the transthalamic pathways described above, which convey information up the cortical hierarchy *via* driver connections, are accompanied by transthalamic pathways that convey modulatory information down the cortical hierarchy (Sherman, 2017). From a cortical perspective, the first could be considered as bottom-up (feedforward, driver) pathways, whereas the second could be considered as top-down (feedback, modulatory) pathways. Making a parallelism with the corticothalamic loop, where feedforward thalamocortical connections are accompanied by feedback CT connections (L6a feedback) to form a corticothalamic loop, the feedforward transthalamic connections could (hypothetically) be accompanied by feedback transthalamic connections to form a transthalamic corticocortical loop. However, there is still no direct evidence that such transthalamic top-down, modulatory pathways exist. Evidence exists that higher order thalamic nuclei provide modulator inputs to first order, primary cortical areas in visual (Purushothaman et al., 2012; Roth et al., 2016), somatosensory (Viaene et al., 2011) and auditory systems (Pardi et al., 2020). Because modulation is provided by a higher order thalamic nucleus to a lower order cortical area, these circuits can be viewed as descending, top-down circuits. Using a combination of optogenetics, whole-cell recordings, behavior, and computational modeling, Pardi et al. (2020) identified an auditory top-down circuit that conveys information about the experience-dependent behavioral relevance of sounds from higher order auditory thalamus (all nuclei surrounding the MGV) to layer 1 in primary auditory cortex (anatomical evidence for this pathway in classical studies: Lorente de No, 1938; Malmierca et al., 2002). Interestingly, synaptic transmission in A1 is in turn modulated by local inhibition acting on GABAB receptors at the presynaptic thalamic terminal. Because higher order thalamic neurons that specifically project to A1 receive inputs from a diversity of higher order cortical areas (e.g., secondary auditory and association cortices), the top-down circuit identified by Pardi et al. (2020) might well be part of a transthalamic feedback circuit that conveys internally generated top-down signals. At the thalamic node, these top-down signals have the chance to be integrated and compared with sensory information conveyed by subcortical inputs to A1-projecting thalamic neurons (e.g., superior colliculus and external cortex of the inferior colliculus; Malmierca et al., 2002; Cai et al., 2019; Pardi et al., 2020).

Such transthalamic feedback pathways would provide modulatory or non-linear context-sensitive effects consistent with the tenets of predictive coding (Friston, 2005; Bastos et al., 2012). In this context, the fact that synaptic transmission of higher order MGB-A1 neurons synapses can be modulated by local inhibition in L1 as Pardi et al. (2020) demonstrated, is particularly interesting because it provides the computational flexibility that feedback connections require to convey predictions in a context-sensitive manner (Bastos et al., 2012; discussed in section Role of Corticothalamic Pathways in the Implementation of Predictive Processing Frameworks).

## Role of Corticothalamic Pathways in the Implementation of Predictive Processing Frameworks

Predictive coding frameworks envisage the brain as a predictive machine that is highly constrained by prior experiences, where signals from the external world shape but do not drive perception. Perception is viewed as an entirely inferential process in which higher brain areas use generative models to make predictions about the outside world and inform lower brain areas of these predictions (Rao and Ballard, 1999; Friston, 2005; Keller and Mrsic-Flogel, 2018). These ideas invert the conventional view of perception as a mostly bottom-up process (Sherrington, 1906), and highlight the importance of top-down, backward pathways that convey predictions to shape sensory-driven activity in lower brain areas (Helmholtz and von, 1867; Craik, 1943).

The canonical computations of predictive processing rely on the circuitry of the cortical column and connections between cortical areas, in which inference is implemented *via* message passing along the cortical hierarchy. However, as we have reviewed in the above sections, the cortex is inextricably linked to the thalamus. Any theoretical framework that ignores the strong link between cortex and thalamus will likely be incomplete (Mumford, 1991; Auksztulewicz and Friston, 2015; Kanai et al., 2015; Rikhye et al., 2018; Carbajal and Malmierca, 2020). In this section, after a brief explanation of how predictive processing is implemented (section Predictive Processing Is Implemented via Hierarchical Perceptual Inference), we will review evidence proposing that the thalamus and the CT pathways are in a key position to dynamically coordinate and contextualize hierarchical inference in cortical hierarchies (section Role of CT Pathways in Coordinating and Contextualizing Inference in Cortical Hierarchies).

### Predictive Processing Is Implemented *via* Hierarchical Perceptual Inference

**Figure 8:**
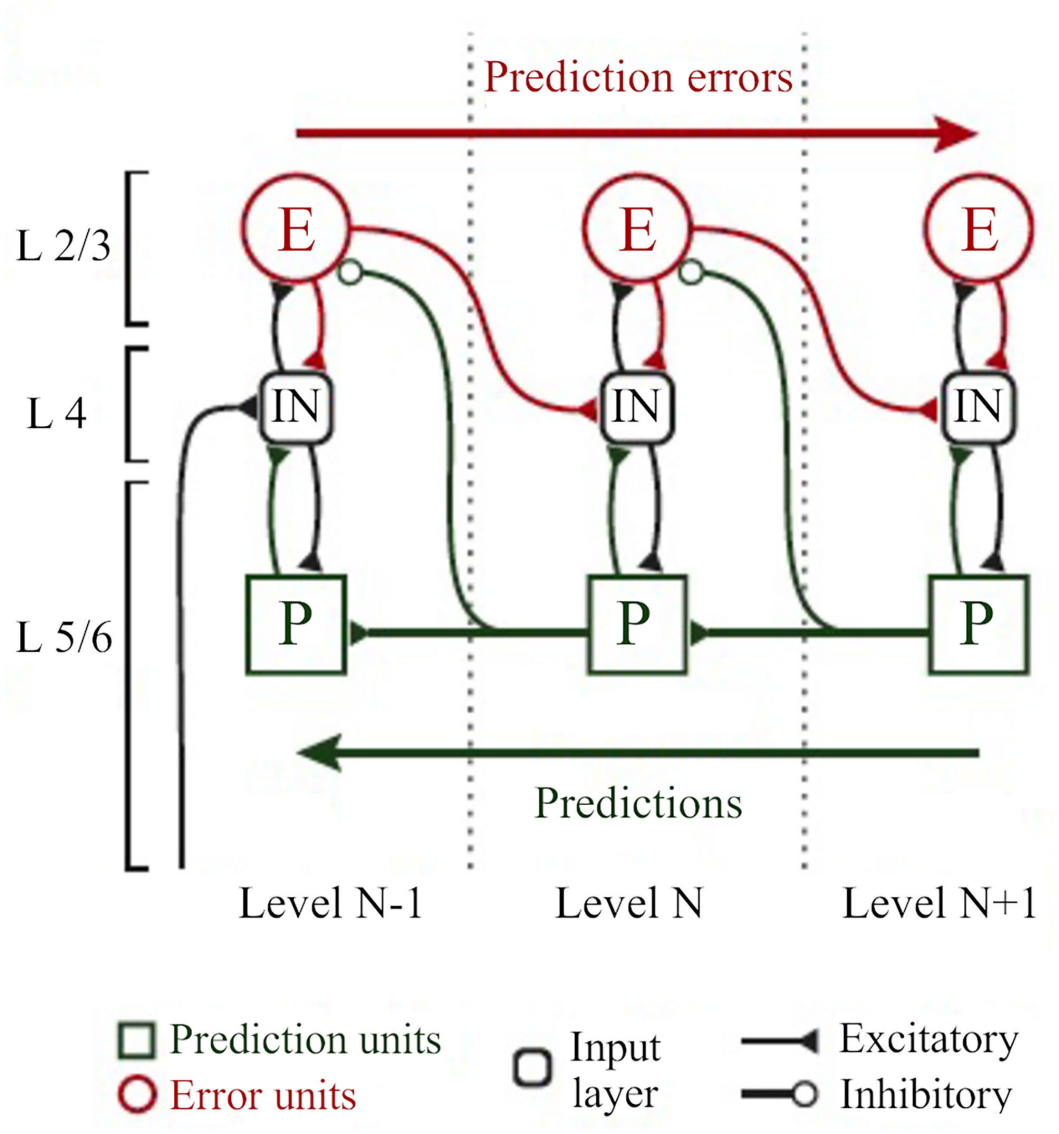
The classical implementation of predictive coding relies on the cortical hierarchy. A simplified hierarchical model based on the classical implementation of predictive coding (Bastos et al., 2012). Vertical dashed lines delimit hierarchically arranged cortical columns (left to right: bottom up). Prediction errors climb up the cortical hierarchy through the feedforward, bottom-up connections, whereas predictions are sent backwards (top-down) to suppress prediction error units of the levels below, via inhibitory connections. Superficial layers (L2/3) above the subcortical input layer (L4) carry prediction errors, whereas deep cortical layers (L5/6) carry predictions. Adapted, with permission, from Heilbron and Chait (2018) and Carbajal and Malmierca (2020).

Higher levels generate the predictions about the pattern of sensory input they should be receiving from the level below across multiple and highly interdependent levels of processing (Bastos et al., 2012; Figure 8). These predictions are the best guesses or Bayesian optimal estimates based simultaneously on both sensory data and prior experience (or beliefs) (Friston, 2005; Bastos et al., 2012). Predictions are sent down the processing hierarchy (*via* feedback, modulatory connections), suppressing congruent incoming sensory signals by “explaining away” whatever differences, or prediction errors, they can by inferring likely causes for the discrepancies (Friston, 2005; Bastos et al., 2012). Only the unexplained components of sensory information pass to higher levels as prediction errors, conveyed by feedforward, driving connections (Friston, 2005; Bastos et al., 2012). This process requires two types of neurons: the neurons encoding the prediction (prediction neurons, associated with activity of pyramidal neurons in deep cortical layers), and the neurons comparing the prediction with the actual bottom-up input (prediction error neurons, associated with activity of pyramidal neurons in superficial cortical layers; Friston, 2005; Bastos et al., 2012; Figure 8). Prediction errors are progressively explained away as they climb each level of the hierarchy, while internal models at higher levels become more global and stable. In other words, the brain continuously exploits these error signals to revise and update its predictive models (new posterior beliefs) in an iterative process to produce better predictions (i.e., minimize prediction errors) with every new piece of reliable sensory evidence (Friston, 2005; Bastos et al., 2012). In this process, the different error signals are not treated equally but their relative influence is adjusted according to their precision. Error precision is estimated as the inverse variance of the prediction error and informs the brain about the relative reliability of that error (Feldman and Friston, 2010; Kanai et al., 2015). High precision errors have greater postsynaptic gain and increased influence, whereas prediction errors with very low precision may be unable to drive postsynaptic responses and lack influence (Feldman and Friston, 2010; Kanai et al., 2015).

### Role of CT Pathways in Coordinating and Contextualizing Inference in Cortical Hierarchies

Predictive processing requires a dual role for backward (top-down) connections (Kanai et al., 2015; Auksztulewicz and Friston, 2016). First, it requires backward connections to exert strong inhibitory influences on their targets—the prediction error neurons on lower hierarchical areas—to suppress or counter their feedforward (bottom-up) driving inputs (Bastos et al., 2012; Auksztulewicz and Friston, 2016; Figure 8). However, because predictive processing requires backward connections to influence neurons in lower areas in a context-sensitive manner, it also needs non-linear (modulatory) inputs to these postsynaptic neurons (Bastos et al., 2012; Auksztulewicz and Friston, 2016). Both roles are fulfilled by L6a CT projections. L6a projections can provide excitatory, modulatory influences on thalamic relay neurons but can also effectively suppress these neurons *via* TRN-mediated inhibition (section L6a Corticothalamic Feedback Forms a Corticothalamic Loop). Since pyramidal cells in deep cortical layers are thought to convey predictions by top-down connections (Bastos et al., 2012; Kanai et al., 2015), it is reasonable to suggest that L6a projections can convey predictions to thalamic neurons (Auksztulewicz and Friston, 2015; Shipp, 2016), as we will discuss in the next section (L6a CT Projection Can Bidirectionally Switch the Excitability of Thalamic Neurons According to Contextual Information and/or Behavioral Demands). This hypothesis is particularly interesting because L6a forms part of corticothalamic loops that embody important functional architecture attributes for predictive coding (Adams et al., 2013): (1) a hierarchical organization with (2) reciprocal connections that are (3) functionally asymmetrical (thalamocortical driver vs. CT modulator). In contrast, L5 rather represents a bottom-up connection because it conveys information from a lower to a higher order region. This is consistent with the fact that L5 has a driving, feedforward nature (Sherman and Guillery, 1998; Llano and Sherman, 2008). For this reason, it is very unlikely that L5 conveys predictions to higher order thalamic nuclei (predictions are conveyed by top-down connections; Friston, 2005). Instead, L5 CT projections can be involved in prediction error related computations (Kanai et al., 2015), as we will discuss in the last section of this review (3.2.2).

#### L6a CT Projection Can Bidirectionally Switch the Excitability of Thalamic Neurons According to Contextual Information and/or Behavioral Demands

L6a CT projection carries the potential to either suppress or facilitate thalamic activity, through the dynamic interaction with GABAergic neurons in the TRN (Pinault, 2004; Crandall et al., 2015). This L6-TRN interaction powerfully controls the thalamus in an activity-dependent manner: low frequency CT activity primarily suppresses thalamic excitability, whereas higher frequency activity shifts CT influence from suppression to enhancement (Crandall et al., 2015; see section 1.1 for synaptic mechanisms). The suppression exerted by low frequency CT activity is compatible with the implementation of predictive coding where feedback predictions are linked to low frequency oscillations that exert a suppressive effect on prediction error units of the level below (Bastos et al., 2012). However, brief periods of sustained activity in L6 can generate a local cortical gamma rhythm that, *via* CT neurons, can quickly shift the cortical effect on thalamic excitability from suppression to enhancement (Crandall et al., 2015).

##### L6a CT Pathway Could Modulate the Gain of Thalamic Error Units According to Their Precision

The ability of L6 projection to bidirectionally switch the excitability of thalamic neurons would make these neurons tunable according to contextual information and/or behavioral demands (Figure 5; Crandall et al., 2015; Guo et al., 2017). For example, suppression of responses to predictable stimuli can coexist with (top-down) attentional enhancement of signal processing (Wyart et al., 2012). Attention has been suggested to optimize precision expectations during hierarchical inference by increasing the gain of neurons encoding prediction errors (Feldman and Friston, 2010; Auksztulewicz and Friston, 2015; Smout et al., 2019), which can override the suppressive influence of top-down expectation (Kok et al., 2012). Similarly, the hippocampus may facilitate both a prediction signal and memory, respectively, by inhibiting neocortical prediction errors or increasing their gain, a mechanism probably dependent on the precision ascribed to prediction error units (Barron et al., 2020).

An enhancement of prediction error can also be useful to facilitate perception to challenging conditions (Auksztulewicz and Friston, 2016), such as to improve detection of low salience visual (Hup et al., 1998) and auditory stimuli (Parras et al., 2017), but also to detect a salient acoustic event in the natural soundscape while performing a competing attentional task (Huang and Elhilali, 2020) or a novel event while listening passively or actively to multiple concurrent acoustic sources (Sohoglu and Chait, 2016). Accordingly, behavioral detection of low salience sounds is improved by the enhancement of tone-evoked cortical responses (Guo et al., 2017). This cortical enhancement is driven by CT influences and arises from an intrathalamic shift in excitatory-inhibitory balance between the auditory thalamus and the TRN (responses are increased in the MGV and reduced in the TRN; Figure 5; Guo et al., 2017). An enhanced thalamic response driven by CT influences can also be a mechanism to compensate for impaired, age-related ascending auditory signals (Cai et al., 2016). Under this scenario, L6 CT projections would have the potential to perform two complementary tasks needed for predictive processing: (1) the ability to convey predictions of perceptual content (first order predictions) that will suppress thalamic neurons encoding prediction errors (*via* TRN inhibition); and (2) the ability to convey predictions of precision (second order predictions) that carry context information in the form of salience or precision ascribed to these prediction errors in order to change their gain (excitatory modulatory effect; higher precision, higher excitation). Whether L6 can perform these tasks is still unknown, but the remarkable ability of L6 to switch the excitatory-inhibitory balance of thalamic neurons (Crandall et al., 2015; Guo et al., 2017) suggests that L6 can provide gain control over thalamic neurons compatible with precision weighting (Kanai et al., 2015).

##### Suppression and Enhancement Coexist in Auditory Thalamic Neurons That Signal a Deviance From Previous Stimulus Context

In higher order auditory thalamus (medial and dorsal subdivisions), suppression of repeated sounds coexists with enhancement to rare, surprising sounds in single neurons, the so-called stimulus-specific adaptation (SSA; Figure 2; Ulanovsky et al., 2003) or neuronal mismatch (Parras et al., 2017). This property is not restricted to the thalamus but is distributed hierarchically from as early as the auditory midbrain to the auditory cortex and prefrontal cortex (some classical studies: AC, Ulanovsky et al., 2003; inferior colliculus, Pérez-González et al., 2005; TRN, Yu et al., 2009; MGB, Antunes et al., 2010; prefrontal cortex, Casado-Román et al., 2020). Classically, SSA is elicited using an oddball paradigm, where two tones that differ in their probability of appearance in a sequence are played (a repeated sound interrupted randomly by a deviant sound; Figure 2). As we progress along the auditory hierarchy, responses to the repeated sound are suppressed, but responses to the deviant are enhanced. Responses to the deviant are maximal in prefrontal cortex (Casado-Román et al., 2020). Because their enhanced responses to surprising sounds cannot be explained solely by bottom-up mechanisms of neural fatigue, these neurons are believed to signal expectancy deviance that can be regarded as a prediction error signal (Parras et al., 2017; Malmierca et al., 2019; Hamm et al., 2021). This enhancement would be the result of modulatory mechanisms exerted by higher order cortical areas (e.g., prefrontal cortex; Hamm et al., 2021) and/or neuromodulatory gain mechanisms compatible with precision-weighting (e.g., dopaminergic neuromodulation; Valdés-Baizabal et al., 2020). For example, in awake rats, this enhancement is stronger when the intensity of stimulation is low. A gain mechanism to facilitate perceptual saliency could underlie this enhancement (Parras et al., 2017; Carbajal and Malmierca, 2018). Although SSA in the MGB is not inherited from the auditory cortex (Figure 2; Antunes and Malmierca, 2011), it is still unknown if this error enhancement is generated by cortical influences, because Antunes and Malmierca (2011) did not use the control sequences used to discern between repetition suppression and error enhancement (for details, see Parras et al., 2017; Casado-Román et al., 2020).

##### The Gain Exerted by the Auditory Cortex on Thalamic Neurons Depends on Their Context-Sensitivity

Although SSA is not inherited from the AC, the AC modulates the excitability of thalamic neurons in a gain-like mechanism that depends on their level of SSA (Figures 2–4; section L6a Corticothalamic Feedback Forms a Corticothalamic Loop; Antunes and Malmierca, 2011). By mainly suppressing high SSA neurons and facilitating non-SSA neurons, the cortex, *via* CT pathways, seems to discriminate between neurons that encode prediction errors (context sensitive, high SSA), and those that perform other type of computations (non-SSA; Antunes and Malmierca, 2011; Figure 4). The suppression of SSA neurons is consistent with an overall inhibitory effect of backward projections (conveying predictions) on prediction error units of the level below (Bastos et al., 2012; Figure 2A). In this case, a precise prediction (repetition of the standard tone) is sent backwards to suppress or “explain away” the sensory prediction error (Auksztulewicz and Friston, 2016). However, some of these prediction error units (high SSA) in the thalamus are facilitated by the cortex (Figure 2B; Antunes and Malmierca, 2011). Hypothetically, this facilitation could be the result of precision-weighting gain mechanisms imposed by CT pathways, probably elicited by the unexpected appearance of a deviant sound (Hamm et al., 2021). However, in our opinion, the discussion of whether SSA neurons are prediction error units in terms of predictive coding is highly speculative. If SSA neurons are indeed error units, they should be accompanied by activity of neurons encoding expectancy in upper hierarchical levels (see Fiser et al., 2016, for an elegant protocol and experiment of this kind). We do not yet have such evidence for SSA (but see Hamm et al., 2021). It remains an open question if the effects observed using the oddball paradigm in single units are a consequence of perceptual expectations.

#### L5 and the Pulvinar at the Crossroads of Contextual Information

The Pulvinar is the multisensory higher order thalamic nucleus per excellence. Because it receives inputs from a diversity of cortical and subcortical areas (e.g., superior colliculus), the pulvinar is uniquely positioned to provide sensory and contextual information to cortical computations (Roth et al., 2016; Jaramillo et al., 2019; Chou et al., 2020). For example, the rodent homolog of the pulvinar (lateral posterior nucleus), conveys diverse contextual information to primary visual cortex neurons that informs these neurons about changes in external motion not predicted by the animal's own actions (Ishiko and Huberman, 2016; Roth et al., 2016). In the auditory system, the lateral posterior nucleus, driven by input from the superior colliculus, provides contextual and cross-modality modulation of A1 responses to enhance the salience of acoustic information (Chou et al., 2020). Specifically, it contributes to the maintenance and enhancement of A1 processing, respectively, in the presence of background noise and threatening visual looming stimuli.

The mouse pulvinar can strongly influence the activity of extrastriate cortical neurons, and particularly of those that project to the striatum and amygdala (Zhou et al., 2018). Because the pulvinar also projects to the striatum and amygdala, Zhou et al. (2018) proposed that the pulvinar can function as a hub linking the visual cortex with subcortical regions involved in coordinating body movement and sensory information (Roth et al., 2016). The fact that the pulvinar exerts strong influences on extrastriate cortical areas challenges the conventional hierarchical view of visual cortical processing in which information transfer to extrastriate areas occurs primarily *via* corticocortical connections (van Essen, 2005; Zhou et al., 2018). However, the rodent pulvinar is not a homogeneous nucleus but has distinct subregions (Nakamura et al., 2015; Foik et al., 2020; Scholl et al., 2021). Based on connectivity and functional properties, Bennett et al. (2019) distinguished three subregions in the mouse pulvinar. The posterior-dorsal subregion is driven by the superior colliculus and responds to looming stimuli and small moving objects, whereas the anterior-ventral region is driven by visual cortex and responds to large stimuli and full-filled motion (Bennett et al., 2019). Their study further suggests that a medial subregion might be involved in transthalamic pathways connecting frontal and associational cortex to visual cortices (Bennett et al., 2019).

The pulvinar provides contextual and cross-modality information to A1 (Chou et al., 2020). However, it certainly processes contextual auditory information in a different way than visual information. Perhaps the analog structure of the visual pulvinar in the auditory system could be the MGM, the principal multisensory subdivision of the auditory thalamus (for a review see Bartlett, 2013). The MGM is intensively connected with auditory and non-auditory cortical areas (e.g., somatosensory cortex; Lohse et al., 2021), other sensory areas (e.g., visual, somatosensory and vestibular), and, like the pulvinar, projects to the amygdala and striatum (for a review see Bartlett, 2013).

**Figure 9:**
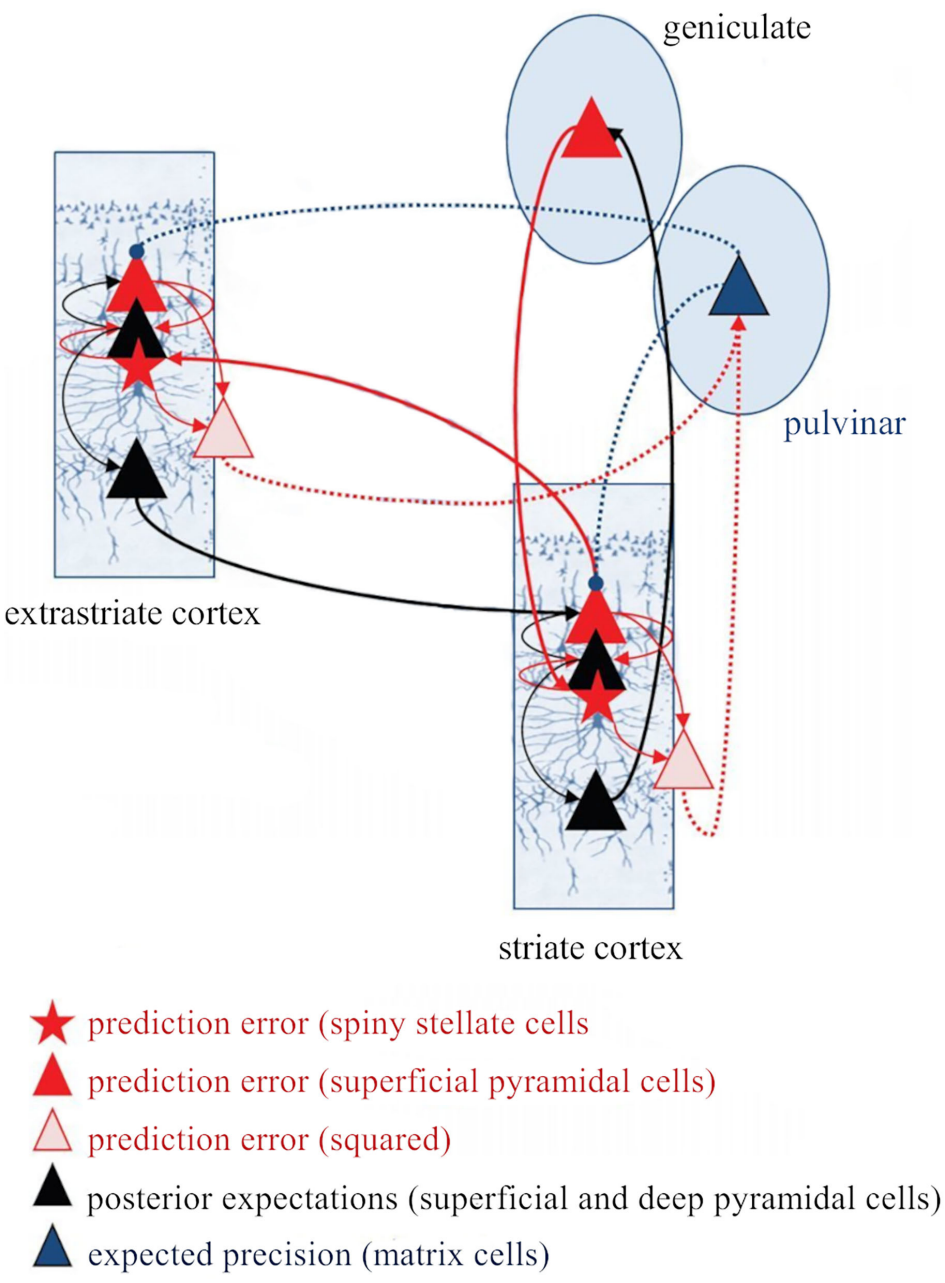
The thalamus and the corticothalamic pathways as key players in the implementation of predictive coding. This scheme, proposed by Kanai et al. (2015) expands the classical implementation of predictive coding [Bastos et al. (2012), Shipp (2016)] as Figure 7 shows, because it incorporates the thalamus and the corticothalamic pathways (both L6a and L5 CT projections) as key players in its implementation. A very interesting aspect of this model is the inclusion of deep pyramidal cells (presumably L5 CT cells) that convey the squared prediction error (second-order forward connections) to enable the pulvinar (matrix cells) to estimate precision. These cells in the pulvinar send back projections to modulate the gain of superficial pyramidal cells in the cortex. Red, forward connections; Black, backward connections; Full lines, first order streams; Dashed lines, second order (precision-related) streams. CT, corticothalamic; L5, cortical layer 5; L6a, cortical layer 6a. Adapted from Kanai et al. (2015).

Kanai et al. (2015) proposed a model were neurons in higher order multisensory thalamic nucleus such as the pulvinar would encode expected precision (Figure 9). This model predicts two different types of streams of information between thalamus and cortex: the first-order streams convey predictions and prediction errors between first order thalamic nucleus and primary cortical areas, *via* L6 CT feedback connections; and the second order streams convey precision-related information between (lower and higher order) cortical areas and higher order thalamic nuclei (Figure 9). This model includes neurons in deep pyramidal cells of (lower and higher order) cortical areas encoding the amplitude of prediction error (squared) that inform, *via* L5 projections, posterior expectations about precision in the cells of the pulvinar (Kanai et al., 2015; Figure 9). Here, it is assumed that these neurons in the pulvinar send a reciprocal feedback to modulate the gain of superficial pyramidal cells in the cortex. This in accordance with Bennett et al. (2019) study in the rodent pulvinar that suggests that L5 participates in reciprocal cortico-pulvinar feedback loops. This model implies that L5 itself forms part of corticothalamic loops that would coordinate precise (direct) corticocortical message passing among different cortical areas (Kanai et al., 2015; Figure 9). Under this scenario, inference about the first order content of perception is attributed to direct corticocortical message passing, whereas parallel transthalamic connections contextualize (second order) corticocortical processing *via* precision-weighted gain control of ascending prediction errors (Kanai et al., 2015).

## Conclusions and Future Directions

The diversity and abundance of CT circuits that tightly link the cortex to the thalamus underscores the functional importance of these circuits to brain function. In recent years, the use of genetic tools to selectively manipulate layer or cell-type specific neuronal populations combined with electrophysiology in the awake animal opened the possibility to directly test the role of CT pathways in behavior (e.g., L6a CT circuit; Guo et al., 2017; Clayton et al., 2021). Importantly, the use of these genetic tools is unraveling a much more complex and diverse CT circuitry than previously evaluated through classical anatomical and physiological studies. The recent discovery that neurons in L6b participate in a CT circuit that differs from both L6a and L5 circuits (Ansorge et al., 2020; Zolnik et al., 2020), just reflects how sparse our knowledge still is about corticothalamic interactions. In the auditory system, specifically, basic physiological studies are lacking regarding L6b and L5 CT circuits. However, new exciting evidence is unraveling the importance of L6a CT feedback in auditory behavior (e.g., perception of complex sounds; Homma et al., 2017).

In this review we have paid particular attention to the transthalamic circuits mediated by L5 CT neurons. The disclosure of their roles, although still incipient, largely contributed to pull apart the old, corticocentric ideas that the thalamus is a passive relay center receiving instructions from the cortex. The existence of transthalamic corticocortical routes challenges the prevailing view that cortical areas communicate with each other exclusively by means of direct, hierarchical corticocortical connections (Felleman and van Essen, 1991; Sherman and Guillery, 2011). Are transthalamic pathways just redundant circuits that convey the same information between cortical areas as the direct corticocortical connections? The radically different anatomical architecture of these routes makes this hypothesis highly unlikely (Usrey and Sherman, 2019). Taking as an example the connection between primary somatosensory cortex and primary motor cortex, the cells of origin of the direct and transthalamic corticocortical routes represent separate populations (Mo and Sherman, 2019). Furthermore, higher order thalamic nuclei (e.g., the pulvinar) not only receive driver inputs from L5 of a lower order cortical area but integrate inputs from a diversity of cortical and subcortical areas (e.g., superior colliculus), offering the possibility of combining sensory signals with the context in which they arise (Roth et al., 2016; Blot et al., 2020; Chou et al., 2020). It is possible that the direct and transthalamic corticocortical routes dynamically interact, and that the recurrent activity resultant from this interaction would allow for the shaping of neural activity necessary for high-level cognitive processes (e.g., language processing; Crosson, 2019).

A remarkable difference between transthalamic and direct corticocortical routes lies on their extrinsic connection targets. Direct projections involve axons without subcortically directed branches (Bourassa and Deschênes, 1995), whereas L5 axons of the transthalamic pathway branch extensively to target extra-thalamic structures in the brainstem and spinal cord as well as higher order thalamic regions and the amygdala (Deschênes et al., 1994; Usrey and Sherman, 2019; Williamson and Polley, 2019). Therefore, the message passing along the cortical hierarchy *via* transthalamic pathways is broadcasted to other subcortical centers (Williamson and Polley, 2019). Many of these centers are motor in nature (e.g., tectum, striatum), suggesting that L5 far-ranging CT axons (including those in primary sensory areas) may be involved in sensorimotor behavior (Prasad et al., 2020).

As descending pathways that enable reciprocal and context-dependent communication between thalamus and cortex, we venture that CT projections are particularly interesting in the context of hierarchical perceptual inference formulations such as those contemplated in predictive processing schemes (Friston, 2005), which so far heavily rely on cortical implementations. However, the thalamus, and particularly higher order thalamus, is in a key position to coordinate and contextualize hierarchical inference in cortical hierarchies (Kanai et al., 2015). An expanded, upgraded version of predictive coding that includes the role of corticothalamic interactions or even (recursive) coupling with other subcortical structures (Auksztulewicz and Friston, 2015; Kanai et al., 2015; Barron et al., 2020), will certainly broaden its explanatory power.

## Author Contributions

FMA and MSM: conceptualization and planning. FMA: writing—original draft and review and editing. MSM: review and editing. Both authors contributed to the article and approved the submitted version.

## Conflict of Interest

The authors declare that the research was conducted in the absence of any commercial or financial relationships that could be construed as a potential conflict of interest.

## Publisher's Note

All claims expressed in this article are solely those of the authors and do not necessarily represent those of their affiliated organizations, or those of the publisher, the editors and the reviewers. Any product that may be evaluated in this article, or claim that may be made by its manufacturer, is not guaranteed or endorsed by the publisher.
